# Ice age unfrozen: severe effect of the last interglacial, not glacial, climate change on East Asian avifauna

**DOI:** 10.1186/s12862-017-1100-2

**Published:** 2017-12-06

**Authors:** Feng Dong, Chih-Ming Hung, Xin-Lei Li, Jian-Yun Gao, Qiang Zhang, Fei Wu, Fu-Min Lei, Shou-Hsien Li, Xiao-Jun Yang

**Affiliations:** 1 Kunming Institute of Zoology, Chinese Academy of Sciences, 32 Jiaochang Donglu Rd., Kunming, Yunnan China; 2 Department of Life Science, National Taiwan Normal University, 88 Ting-chou Rd., Sec. 4, Taipei, 116 Taiwan; 3 Biodiversity Research Center, Academia Sinica, Taipei, Taiwan; 4 Guangdong Institute of Applied Biological Resources, Guangzhou, 510260 China; 5 Key Laboratory of Zoological Systematics and Evolution, Institute of Zoology, Chinese Academy of Sciences, 1 Beichen West Rd., Chaoyang District, Beijing, 100101 China

**Keywords:** Coalescent simulations, Ecological niche modeling, East Asian birds, Pleistocene climate change, Last interglacial period, Last glacial maximum

## Abstract

**Background:**

The glacial-interglacial cycles in the Pleistocene caused repeated range expansion and contraction of species in several regions in the world. However, it remains uncertain whether such climate oscillations had similar impact on East Asian biota, despite its widely recognized importance in global biodiversity. Here we use both molecular and ecological niche profiles on 11 East Asian avian species with various elevational ranges to reveal their response to the late Pleistocene climate changes.

**Results:**

The ecological niche models (ENM) consistently showed that these avian species might substantially contract their ranges to the south during the Last Interglacial period (LIG) and expanded their northern range margins through the Last Glacial Maximum (LGM), leading to the LGM ranges observed for all 11 species. Consistently, coalescent simulations based on 25–30 nuclear genes retrieved signatures of significant population growth through the last glacial period across all species studied. Climate statistics suggested that high climatic variability during the LIG and a relatively mild climate at the LGM potentially explained the historical population dynamics of these birds.

**Conclusions:**

This is the first study based on multiple species and both lines of ecological niche profiles and genetic data to characterize the unique response of East Asian biota to late Pleistocene climate. The present study highlights regional differences in the evolutionary consequence of climate change during the last glacial cycle and implies that global warming might pose a great risk to species in this region given potentially higher climatic variation in the future analogous to that during the LIG.

**Electronic supplementary material:**

The online version of this article (10.1186/s12862-017-1100-2) contains supplementary material, which is available to authorized users.

## Background

Recent glacial cycles have been well recognized as one of the major forces shaping patterns of global biodiversity [1–4]. It is widely perceived that species responded to the cyclical climatic changes in the Pleistocene by the repeated “expansion-contraction” of their ranges during the alternating glacial and interglacial periods [2]. For example, advancing ice sheets in the Pleistocene drove the contraction of temperate biota to refugia and the expansion of polar biota in glaciated regions such as Europe [1, 2], North America [5] and Antarctica [6]. Conversely, in unglaciated regions increasing aridity due to global cooling during glacial maxima led to range contraction in mesic-adapted species and range expansion in arid-adapted species, as reported in South America and Africa [7], Australia [8] and Sundaland [9]. It has been argued that the alternative warmer and wetter climate during interglacial periods caused the range changes in these regions into the reverse [3, 10]. However, whether climate oscillations during the Pleistocene lead to cyclical range changes in the biota of East Asia, a biodiversity hotspot [11], has not been comprehensively assessed.

East Asia was not covered by ice sheets during the entire Pleistocene [12]. Palynological data indicate a southward contraction of subtropical vegetation at the Last Glacial Maximum (LGM; 19–26 thousand years ago, Ka) [11]. Despite confirmation by several ecological niche modeling (ENM) studies [13–15], other recent studies failed to find any significant impact of the LGM on the distributions of several plant and animal species in East Asia [16–22]; rather, some studies observed substantial range contraction during the warmer Last Interglacial period (LIG, ~112–132 Ka) [18–24]. Given that severe range retraction can cause dramatic reductions in population [25], one might expect small effective population sizes (*N*_*e*_) in East Asian species because of either LGM or LIG contraction. By contrast, several genetic analyses found confounding effects, e.g. with demographic growth under a range contraction scenario [13] or with constant population size through Pleistocene glaciations [26]. Thus, it was hypothesized that Pleistocene glaciations might have had a minor impact on East Asian organisms [26]. However, such discrepancies may have been caused by limitations in study design, for example, with low power to detect population decline (e.g. bottleneck) based on limited number of loci [27], such as a single maternally inherited locus (e.g. mitochondrial or chloroplast DNA) [16, 28] or few, if any, nuclear genes [17, 26]. In addition, previous studies only focused on one or few species precluding a comprehensive understanding of community level dynamics. Therefore, analyses based on multiple species and multiple loci are required to robustly test for demographic dynamics of biota in this region [29].

In the present study, we used ecological niche profiles and genetic data to examine the impact of the last interglacial-glacial cycle on 11 non-migrant bird species common in subtropical East Asia. These birds can be categorized into three classes with different elevational distribution ranges reflecting the breadth of their habitats (see details in Additional file 1: Table S1): five elevational generalists (elevational range: 0–4000 m; *Aegithalos concinnus*, *Cettia fortipes*, *Leiothrix lutea*, *Pomatorhinus ruficollis*, and *Stachyridopsis ruficeps*), four highland specialists (1000 - 4000 m; *Fulvetta ruficapilla*, *Lioparus chrysotis*, *Parus monticolus*, and *Yuhina diademata*) and two lowland specialists (mainly 0 - 1500 m; *Alcippe morrisonia* and *Spizixos semitorques*). Given that their ranges are mostly confined to the subtropics (south of 34° N), these birds are likely to have been sensitive to historical environmental changes, presenting an ideal system to examine biological responses to late Pleistocene climate in East Asia. Understanding their responses to historical climate change is also critical to predict the future fate of the East Asian biota under the ongoing global warming.

Because climate plays a primary and necessary role in species’ range limitation [30], we first used ENM analyses to examine whether the birds’ habitats were subject to regular glacial contraction and interglacial expansion during the last glacial cycle (i.e. the LIG, LGM and present day). Range dynamics (expansion or contraction) resulting from climate change are expected to have had pronounced genetic consequences in populations [25, 31]. So we further performed coalescent analyses based on 25–30 nuclear loci for each species to examine whether the last glaciation cycle had impacted population genetic variation and demographic dynamics of the 11 East Asian birds.

## Methods

### Ecological niche modeling

We used ENM analyses to model range dynamics between the LIG, LGM and Present. Raw occurrence records for each of the 11 species (see details in Additional file 2), following the taxonomy in the *Handbook of the Birds of the World (HBW)* series, were compiled from the eBird Basic Dataset (http://ebird.org/content/ebird, accessed Aug 2015) and China Bird Report (http://www.birdreport.cn, accessed Aug 2015), and outliers from their known distributions were removed. To avoid spatial autocorrelation, the retained records were then rarefied at a 5-km spatial resolution under a south Asian continent equidistant conic projection in SDMtoolbox 1.1c [32] (see dataset in Additional file 3), roughly equivalent to the resolution of climatic layers (e.g. 2.5 arc-minute resolution). Climate grids for the present and LGM (under the Community Climate System Model) were downloaded from the WorldClim website [33] (http://www.worldclim.org) with 2.5 arc-minute resolutions. The LIG model was initially obtained with a 30 arc-second resolution and then resampled to 2.5 arc-minute using a “NEAREST” algorithm using SDMtoolbox. The reasonability of study area extent is crucial for accuracy of ENM analyses and is difficult to determine [34]. Given present distributions, we set an enlarged area exceeding the mainland Oriental Region [35], sufficiency of which was verified by whether or not embracing the species’ distribution changes across historical climate changes.

To reduce model overfitting associated with factor autocorrelation, highly correlated (*r* > 0.90) bioclimatic variables were identified and finally 10 variables were reserved, namely Bio_2 (mean diurnal range in temperature), Bio_3 (isothermality, monthly/ annual temperature range), Bio_5 (maximum temperature of warmest month), Bio_7 (temperature annual range), Bio_11 (mean temperature of coldest quarter), Bio_14 (precipitation of driest month), Bio_15 (precipitation seasonality, coefficient variation), Bio_16 (precipitation of wettest quarter), Bio_18 (precipitation of warmest quarter) and Bio_19 (precipitation of coldest quarter). In addition, winter records for species with significant seasonal migration (e.g. *S. semitorques* and *C. fortipes*, see details in Additional file 1: Table S1) were excluded from the downstream analyses to improve prediction accuracy. The present-day ENM for each species was created and then extrapolated to palaeoclimatic layers at the LGM and LIG in MAXENT 3.3.3 k [36]. Model parameters were trained with 75% of the occurrence records randomly sampled for each species and test by the other 25%, except that for species (*F. ruficapilla* and *L. chrysotis*) with fewer occurrence records (e.g. <100) all data were used to train the models (see details in Additional file 1: Table S3). Two options in MAXENT were applied to reduce model over-prediction, with the maximum training sensitivity plus specificity (MTSS) threshold and the fade-by-clamping (i.e. to mitigate clamping issues during extrapolation among nonanalogous climates) procedure. Model performance was evaluated with the statistics of the area under the receiver operating characteristic curve (AUC).

### Climatic statistics

To characterize the climate of the last glaciation cycle, we compared climatic variations among the LIG, LGM and present day, and those across latitudes at the LIG, by extracting bioclimatic values based on a spatially rarefied occurrence dataset combined from all 11 birds studied. All the occurrence records across species were first combined and then rarefied by a 5-km distance, to approximate one point in one grid. Based on these points, values of the 10 bioclimatic variables used in above ENM analyses were extracted using DIVA-GIS 7.5 [37] and the significance of differences was calculated with a paired-sample T-test.

### Coalescent simulations

Coalescent simulations were conducted to uncover demographic changes through time. For each species studied, 17–34 (typically 25–34, except 17–19 for *F. ruficapilla*, *S. semitorques,* and *P. monticolus*) individuals were sampled, a number sufficient to accurately estimate population size changes [38].

Genomic DNA was extracted using conventional proteinase *K* digestion and phenol/chloroform extraction [39]. For each species, totally 25–31 nuclear loci (including introns and exons, see details in Additional file 1: Table S2) were amplified and sequenced for all samples using published primers and protocols [40–42]. The DNA sequences were compiled in SeqMan 7.1.0 (DNAStar Inc., Madison, WI) and were aligned in Clustal X 1.83 [43]. Nuclear genotypes were phased using PHASE 2.1.1 [44] with five runs for 1000 iterations. Genotypes with low phasing probability (< 0.60) were excluded from all subsequent analyses, considering their uncertainties [45]. Recombination within each locus was detected by three non-parametric methods (RDP, GENECONV and MAXCHI) with RDP 3.44 [46]. Evolutionary neutrality was tested using Tajima’s *D* [47] and Fu and Li’s D* [48] statistics in DnaSP 5.10.01 [49].

Bayesian coalescent inference of demographic history was conducted using BEAST 1.8.2 [50] with model selection. For each species, five demographic models were considered, including four parametric models (constant, expansion, exponential and logistic growth) and the flexible Bayesian Skygrid model [51]. The margin likelihoods of each model were estimated using both stepping-stone and path sampling methods [52] based on three replicates of 500 million MCMC generations with the HKY substitution model and strict molecular clocks, and compared by *log Bayes factor* with values larger than three indicating a strong preference of one model against others [53]. Recent mounting evidence has suggested time-dependent rates of molecular evolution with faster rates over shorter time frames than what are calibrated by phylogenetic divergences, in some cases by half [54] to an order of magnitude [55, 56]. In present study, the BEAST analyses were first conducted with a fixing mean substitution rate across genes of 3.3 × 10^−9^ substitutions per site per year (s/s/y) for Passeriformes [57], and then rescaled using 5-fold (i.e. 1.65 × 10^−8^ s/s/y) and 10-fold (3.3 × 10^−9^ s/s/y) faster rates, respectively, to test the sensitivity of the prior settings. Convergence for each run was visually inspected and diagnosed with an effective sample size >200 in Tracer 1.6 [58]. Final plotting of the best-fit demographic model for each species was generated based on the combined MCMC generations after discarding the first 10% as burn-in.

In addition, we tested population expansions using a Bayesian algorithm implemented in LAMARC 2.1.8 [59] by inferring exponential population growth rates. Positive growth rates would indicate a growing population, while negative values suggest population shrinkage. We performed one short MCMC run (1000 samples, 100 steps as interval and 10,000 samples as burn-in) followed by a longer one (10,000 samples, 100 steps as interval and 10,000 samples as burn-in) for each species with default model priors. To improve search performance, a heating scheme was used with one cold and three heated chains with default temperature settings. Convergence on the parameter estimates was visually inspected in Tracer 1.6 with an effective sample size >200.

## Results

**Fig. 1:**
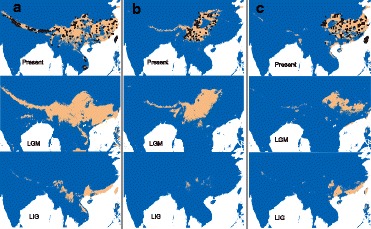
The three patterns of ecological niche models (ENMs) and distribution dynamics for birds studied in the present study. (**a**) *Aegithalos concinnus*, (**b**) *Yuhina diademata* and (**c**) *Spizixos semitorques.* For each species, three time points were considered, i.e., the present day (Present), last glacial maximum (LGM; ~26–19 thousand years ago, Ka), and last interglacial period (LIG; ~112–132 Ka). Black dots show species occurrence records rarefied at a 1-km spatial resolution. To avoid over-prediction, the ENMs were transformed to binary (presence/ absence) distribution by a threshold of the maximum training sensitivity plus specificity (MTSS), with detailed logistic values specified in Additional file 1: Table S3

After data filtering and spatial rarefication, 77–532 occurrence records for each bird were used in ENM reconstructions (see details in Additional file 1: Table S3). The resulted ENM for each species well encompassed historical distribution dynamics, indicating the reasonability of study area setting [34]. With high AUC scores (0.924–0.975, see details in Additional file 1: Table S3), the ENM analyses for all species clearly showed that the hindcasted area of suitable habitats for the 11 birds at the LGM were similar to those of the present day, whereas each species contracted its range southward to low-latitude areas at the LIG but with three different patterns (Fig. 1 and Additional file 4: Figure S1). That is, (1) the highland specialists contracted to the southern Heng-Duan Mountains in southwest China, (2) the lowland specialists retreated to coastal regions of southeast China and (3) the elevational generalists tended to survive at both of the two regions. Generalists had larger distribution ranges than specialists throughout the last glaciation cycle.

**Fig. 2:**
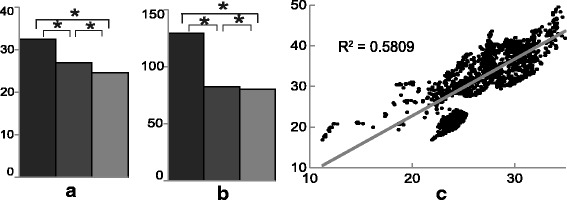
Histograms indicating climatic comparisons. (**a**) Temperature annual range (Bio_7), (**b**) Precipitation seasonality (Bio_15) among LIG (black), LGM (dark grey) and Present (light grey) and (**c**) relations of temperature variations (Bio_7) (*y* axis) and latitudes (*x* axis) at LIG. The means of each variable was calculated based on 1506 occurrence records after spatial filtration at 1 km^2^ of all the combination records of 11 birds. The temperature data are in centigrade, precipitation is in millimeters and the latitudes are in degrees. The asterisk (*) indicates *P* ≤ 0.001 calculated with a paired-samples T test

When compared to that at either the LGM or present, climate summary statistics during the LIG (Fig. 2a and b) showed both enlarged seasonal temperature and precipitation, being warmer in summers, wetter in wet seasons, colder in winters and drier in dry seasons (Additional file 5: Figure S2). In addition, the climatic variations increased with latitude in this region at the LIG (Fig. 2c and Additional file 6: Figure S3).

For the sequences of all the loci generated in the present study, no intra-locus recombination was detected. For each species, 0–3 loci with significant values both in Tajima’s *D* and Fu and Li’s D* statistics (see details in Additional file 1: Table S2) were conservatively excluded from subsequent analyses, despite the difficulty in distinguishing between demographic change and natural selection as the cause for the observed non-neutrality. Finally, a final dataset of 25–30 evolutionarily independent and neutral loci were employed for each bird species (Table 1 and Additional file 1: Table S2). All sequences generated in the current study were deposited in GenBank (accession numbers: KY434671-KY435354, KY437041-KY437069, KY597864-KY604704) and also provided in Additional file 7.

**Table 1:** Summary information for species studied, including species names, number of sampled individuals and loci, as well as log marginal likelihood for each demographic model. Best fitting models with *log Bayes factor* generally larger than 3 against others are highlighted in bold

Species	No. Inds^a^	No. Loc^b^	Output of LAMARC analyses	Output of BEAST analyses^d^				
				Constant size	Exponential growth	Logistic growth	Expansion growth	Bayesian Skygrid
			*g* (95%CI)^c^					
*Aegithalos concinnus*	29	25 (25)	873.09 (623.44, 919.20)	−18,381.02/ −18,387.41	−18,337.58/ −18,345.80	−18,335.44/ −18,342.76	−18,337.65/ −18,344.41	**−18,328.25/** **−18,337.20**
*Alcippe morrisonia*	31	26 (26)	445.00 (213.07, 808.07)	−21,925.05.24/ −21,761.34	−21,321.76/ −21,334.32	**−21,310.73/** **−21,321.83**	−21,329.74/ −21,340.07	−21,319.53/ −21,330.55
*Cettia fortipes*	28	26 (25)	864.57 (657.38, 914.89)	−20,866.90/ −20,871.83	−20,816.09/ −20,822.49	**−20,810.44/** **−20,817.28**	−20,813.41/ −20,821.15	−20,818.79/ −20,825.96
*Fulvetta ruficapilla*	17	27 (25)	254.87 (−288.12, 835.27)	−23,164.78/ −23,166.04	−23,157.21/ −23,159.99	−23,158.79/ −23,160.72	**−23,154.82/** **−23,156.59**	−23,165.93/ −23,167.89
*Leiothrix lutea*	26	27 (25)	889.45 (721.87, 930.99)	−19,430.79/ −19,436.22	−19,371.60/ −19,379.16	**−19,366.81/** **−19,374.85**	−19,373.72/ −19,381.19	−19,371.27/ −19,378.37
*Lioparus chrysotis*	25	26 (26)	691.00 (119.92, 867.68)	−22,793.63/ −22,796.47	−22,789.32/ −22,792.89	**−22,777.02/** **−22,781.68**	−22,781.52/ −22,786.17	−22,558.18/ −22,559.17
*Parus monticolus*	19	28 (26)	839.81 (−24.42, 896.38)	−22,914.29/ −22,916.07	−22,902.45/ −22,905.75	**−22,899.97/** **−22,902.93**	−22,903.01/ −22,905.79	−22,906.41/ −22,910.13
*Pomatorhinus ruficollis*	34	27 (25)	586.95 (293.02, 854.46)	−19,062.06/ −19,070.21	−19,037.46/ −19,050.57	−19,030.12/ −19,042.18	−19,033.39/ −19,044.01	**−19,024.95/** **−19,037.02**
*Spizixos semitorques* ^e^	26	26 (26)	835.62 (259.03, 881.86)	−19,240.68/ −19,242.86	**−19,222.16/** **−19,224.94**	**−19,221.68/** **−19,223.76**	−19,228.76/ −19,231.84	−19,229.73/ −19,233.30
*Stachyridopsis ruficeps*	31	31 (30)	842.18 (431.79, 890.58)	−25,752.35/ −25,763.90	−25,716.61/ −25,729.62	−25,707.27/ −25,722.91	**−25,701.14/** **−25,715.47**	−25,718.02/ −25,734.83
*Yuhina diademata*	32	25 (25)	749.45 (240.43, 880.94)	−24,669.52/ −24,677.78	−24,653.90/ −24,662.24	**−24,643.13/** **−24,654.81**	−24,654.13/ −24,664.42	−24,657.12/ −24,665.48

^a^number of individuals sampled for each species

^b^number of loci sampled for each species with that finally used in brackets

^c^the most probable estimates of population growth rate for each species with 95% credibility intervals (CI) in brackets

^d^log marginal likelihood estimates for different species and coalescent models, estimated using path sampling and step-stone procedures

^e^this species has two equivalently best-fitted and general similar models, and the model with smallest log marginal likelihood (e.g., logistic growth) was shown in Fig. 2

**Fig. 3:**
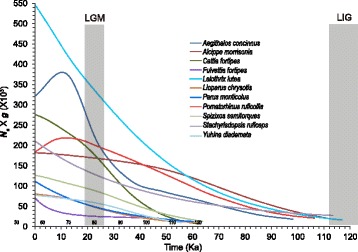
Demographic reconstruction of the most favored models for each bird studied at 5-fold molecular rates. The colored plot lines represent median posterior estimates of the product of effective population size (*N*_*e*_) and generation time (*g*). Times are in thousands of years before present (Ka). Timeframes are marked by grey areas. LIG, last interglacial period, ~112–132 Ka; LGM, last glacial maximum, ~19–26 Ka

The favored models of multi-locus coalescent analyses using BEAST (Table 1) supported an overall post-LIG population growth leading to a ~3–32-fold increase in their effective population sizes up to the present day (see details in Additional file 8: Figure S4). Employment of different molecular rate levels resulted in distinct time spans for the observed population growths but with a consistent pattern of demographic growth from LIG to LGM (Fig. 3 and Additional file 8: Figure S4). This history of population expansion was further supported by large positive exponential population growth rates (254–873; Table 1) estimated using LAMARC. Across the 11 birds, continuous population growth started in two distinct phases. For example, with 5-fold faster molecular rates, elevational generalists except *C. fortipes* and one lowland specialist (*A. morrisonia*) underwent population growth around 98–116 Ka, right after the LIG; whereas the other six species (the four highland and one lowland specialist and one generalist) grew around 40–64 Ka, at the middle of the last glacial period (~12–112 Ka).

## Discussion

In the present study, both our range and demographic analyses imply the contraction of the East Asian subtropical birds’ populations at the LIG and a subsequent expansion through the LGM to the present. The results suggest that the glacial maximum might only have a minor impact on the demography of East Asian organisms in contrast to the potential severe effect of the last interglacial climate. The concordant patterns of change in the multiple species allow us to make a more general picture about late Pleistocene range shifts in East Asian avifauna, which was alluded to by previous studies based on one or a few species [17, 23, 24].

The response pattern suggested by our present study stands in contrast to the prevailing view of cyclical range shifts for temperate species, which postulates glacial contraction and post-glacial expansion. These results also contradict the palynological analysis suggesting an LGM contraction in East Asia. The power of palynological reconstructions might be compromised by the scarcity of pollen-fossil data, a problem prevalent in unglaciated regions in the Pleistocene [4]. For example, few pollen fossils from the LGM have been obtained in subtropical China [60].

The LIG has long been recognized as a climatic optimum for most mesic-adapted species [61], and fossil records suggest similar avifaunas between the LIG and the current interglacial period in temperate regions of Europe and North America [62]. However, our analyses revealed species’ range and demographic contraction at this time, suggesting that the East Asian climate at the LIG had some significant evolutionary consequences. Climate summary statistics showed larger seasonal climatic variability at the LIG than the LGM or present (Fig. 2a and b), which increased with latitude in this region at the LIG (Fig. 2c and Additional file 6: Figure S3). This climatic scenario is consistent with results from recent simulation studies of LIG climate based on orbit parameters, which also showed that the larger temperature seasonality over East Asia during LIG might have resulted from seasonal fluctuations in incoming solar radiation [63]. Given the nature of the limited tolerance of subtropical species to climatic variations [64], the increased LIG climate seasonality at higher latitudes in East Asia might have caused the predicted southern contraction and declines in the study species’ population during the LIG.

The three LIG southern contraction patterns among species (Additional file 4: Figure S1) might be associated with different environmental requirements (Additional file 1: Table S1). The asynchrony at population growth (Fig. 3 and Additional file 8: Figure S4) might illustrate a difference in species’ responses to habitat availability during the transition from the LIG into the last glacial period, reflecting the possibility that their specific climatic and environmental tolerances were associated with the breadth of their ecological niches [3]. For example, precipitation in the warmest quarter (Bio_18) is the most influential bioclimatic variable in present-day ENM prediction for all five of the species with early population growth, but makes a secondary contribution for the other species with late growth (Additional file 1: Table S3).

## Conclusions

Our study demonstrates that the conventional views of glacial contraction and interglacial expansion for mesic-adapted species cannot be applied to all the earth’s climatic zones. In East Asia, subtropical avifauna showed narrower distribution and smaller *N*_*e*_ at the LIG and then bounced back to a larger LGM range similar to the current one. The asynchronous range dynamics among organisms in different ecozones might have led to variable evolutionary consequences. For example, at the LGM temperate species might have retreated to isolated refugia facilitating population divergence and speciation [1, 65], while subtropical species might have expanded their ranges with increasing gene flow and obscuring differentiation among populations. Therefore, a comprehensive assessment of regional differences is critical to test historical biogeographic hypotheses globally.

This study also suggests that high climatic variation, rather than high mean temperatures, explain the potential range contraction of subtropical birds at the LIG. The LIG climate might reflect the near future climate characterized by increased climatic variation under the impact of global warming [61, 64]. Accordingly, we can predict that a continuing trend of climate warming will pose a serious threat to subtropical biodiversity and thus justifies more conservation attention.

## Additional files

Additional file 1: Table S1. Detailed information for each species. **Table S2.** Summary of key statistics for sequences of each studied species. **Table S3.** Detailed information for ENM analyses. This includes estimates of model fit and the relative percentage contributions of the climatic variables to the ENM models for each species studied. (DOCX 68 kb)

Additional file 2: Raw occurrence dataset for the 11 birds studied. (XLS 403 kb)

Additional file 3: Occurrence data after rarefaction for the 11 birds studied. (XLS 266 kb)

Additional file 4: Figure S1. Ecological niche models (ENM) and distribution dynamics. (A) *Aegithalos concinnus*, (B) *Cettia fortipes*, (C) *Leiothrix lutea*, (D) *Pomatorhinus ruficollis*, (E) *Stachyridopsis ruficeps*, (F) *Alcippe morrisonia*, (G) *Spizixos semitorques*, (H) *Fulvetta ruficapilla*, (I) *Lioparus chrysotis* and (J) *Parus monticolus* (K) *Yuhina diademata*. For each species, three time points were considered, i.e., the present day (Present), last glacial maximum (LGM; ~26–19 thousand years ago, Ka), and last interglacial period (LIG; ~132–112 Ka). Black dots show species occurrence records rarefied at a 5-km spatial resolution. To avoid over-prediction, the ENMs were transformed to binary (presence/ absence) distribution by a threshold of the maximum training sensitivity plus specificity. (PDF 9168 kb)

Additional file 5: Figure S2. Histograms indicating comparisons of ten climatic variables among LIG, LGM and Present. The means of each variable was calculated based on 1506 occurrence records after spatial filtration at 5 km of all the combination records of 11 birds. The temperature data are in centigrade (°C) and precipitation is in millimeters (mm). The asterisk (*) indicates *P* ≤ 0.001 calculated with a paired-samples T test. The results show larger seasonal climatic variability at the LIG than the LGM or the present, being warmer in summers (Bio_5), colder in winters (Bio_11), drier in dry seasons (Bio_14) and wetter in wet seasons (Bio_16). (PDF 407 kb)

Additional file 6: Figure S3. Relations between climatic indices (*y* axis) and latitudes (*x* axis) at LIG. The temperature data are in centigrade, precipitation is in millimeters and the latitudes are in degrees. The results show that annual temperature variations increased while winter precipitation and temperature decreased toward the north at the LIG. (PDF 1576 kb)

Additional file 7: The raw sequence data generated in the study. (TXT 5410 kb)

Additional file 8: Figure S4. Demographic reconstruction of the most favored models for each bird studied at original (a) and 10-fold (b) molecular rates. The colored plot lines represent median posterior estimates of the product of effective population size (*N*_*e*_) and generation time (*g*). Times are in thousands of years before present (Ka). Timeframes are marked by grey areas. LIG, last interglacial period, ~112–132 Ka; LGM, last glacial maximum, ~19–26 Ka. (PDF 476 kb)

## Abbreviations

AUC: The area under the receiver operating characteristic curve
ENM: Ecological niche model
Ka: Thousand years ago
LGM: Last glacial maximum
LIG: Last interglacial period
MTSS: The maximum training sensitivity plus specificity
*N*_*e*_: Effective population sizes
