# Supplementary material for: Ice age unfrozen: severe effect of the last interglacial, not glacial, climate change on East Asian avifauna
Source: BMC Evol Biol. 2017 Dec 6;17:244. doi: 10.1186/s12862-017-1100-2 (PMC5719578; doi:10.1186/s12862-017-1100-2)
Supplement: Supplementary file 1 — Detailed information for each species. Table S2. Summary of key statistics for sequences of each studied species. Table S3. Detailed information for ENM analyses. This includes estimates of model fit and the relative percentage contributions of the climatic variables to the ENM models for each species studied. (DOCX 68 kb) [file 12862_2017_1100_MOESM1_ESM.docx]

**Title: Ice age unfrozen: Severe effect of the last interglacial, not glacial, climate change on East Asian avifauna**

Feng Dong^1,2,#^, Chih-Ming Hung^3,#^, Xin-Lei Li^1^, Jian-Yun Gao^1^, Qian Zhang^4^, Fei Wu^1^, Fu-Min Lei^5,*^, Shou-Hsien Li^2,*^, Xiao-Jun Yang^1,*^

^1^ State Key Laboratory of Genetic Resources and Evolution, Kunming Institute of Zoology, Chinese Academy of Sciences, Kunming 650223, China;

^2^ Department of Life Science, National Taiwan Normal University, Taipei 116, Taiwan;

^3^ Biodiversity Research Center, Academia Sinica, Taipei, Taiwan

^4^ Guangdong Institute of Applied Biological Resources, Guangzhou 510260, China

^5^ Key Laboratory of Zoological Systematics and Evolution, Institute of Zoology, Chinese Academy of Sciences, Beijing 100101, China

**Additional file 1** Supplementary tables

**Table S1** Detailed information for each species.

| Species | sampling localities | categories | breeding altitudes | habitats | seasonal movement | literature |
| --- | --- | --- | --- | --- | --- | --- |
| *Aegithalos concinnus* | Hanzhong, shaanxi  Xiangxi, Hunan  Luan, Anhui | altitudinal generalist | 75-3960m | mainly edge habitats in broadleaf forest, bushy habitats | Limited | [1] |
| *Cettia fortipes* | Hanzhong, shaanxi  Xiangxi, Hunan  Luan, Anhui  Zunyi, Guizhou  Meishan, Sichuan | altitudinal generalist | 300-3300m | mainly bushy and grassy habitats | Short-distance altitudinal migration, some birds disperse into higher or lower altitudes during end October to April in non-breeding season. | [2] |
| *Leiothrix lutea* | Hanzhong, shaanxi  Xiangxi, Hunan | altitudinal generalist | 75-3400m | mainly thick undergrowth in open forest, forest edge and bushy habitats | Limited | [3] |
| *Pomatorhinus ruficollis* | Luan, Anhui  Xiangxi, Hunan | altitudinal generalist | 200-3400m | mainly undergrowth in open forest, bamboo, and bushy habitats | Limited | [3] |
| *Stachyridopsis ruficeps* | Hanzhong, shaanxi  Luan, Anhui | altitudinal generalist | 200-3200m | mainly broadleaf forest and bushy habitats | Limited | [3] |
| *Alcippe morrisonia* | Hanzhong, shaanxi  Luan, Anhui | lowland specialist | 50-2500m | Mainly low- to mid-altitude montane broadleaf evergreen forest and bushy habitats | Limited | [3] |
| *Spizixos semitorques* | Luan, Anhui  Hanzhong, shaanxi  Qiandongnan, Guizhou | lowland specialist | plain-1700m | mainly open woodland, secondary growth, and bushy and grassy habitats | Limited | [4] |
| *Fulvetta ruficapilla* | Hanzhong, shaanxi | highland specialist | 1250-2800m | mainly broadleaf evergreen oak forest and sometimes bushy habitats | Limited | [3] |
| *Lioparus chrysotis* | Hanzhong, shaanxi  Zunyi, Guizhou  Meishan, Sichuan,  Yaan, Sichuan  Dujiangyan, Sichuan | highland specialist | 1100-3050m | mainly dense undergrowth in evergreen and temperate forests | Limited | [3] |
| *Parus monticolus* | Hanzhong, shaanxi  Zunyi, Guizhou  Qiandongnan, Guizhou | highland specialist | Typically 1100-3960m, but 220-1580m in SE Asia, 800-2700 in Taiwan | diverse habitats mainly in montane subtropical and temperate forest | Short-distance altitudinal migration, some birds disperse into higher or lower altitudes during October to March in non-breeding season. | [4] |
| *Yuhina diademata* | Hanzhong, shaanxi  Xiangxi, Hunan | highland specialist | 1100-3600m | mainly bushy habitats and sometimes open broadleaf evergreen forest | Limited | [3] |

**References**

1. Harrap S. Family Aegithalidae (Long-tailed Tits). In; Hoyo JD, Elliott A, Christie D, editors. Handbook of the Birds of the World, 13. Barcelona: Lynx Edicions; 2008. pp 96.
2. Bairlein F, Alström P, Aymi R, Clement P, Dyrcz A, Gargallo G, et al. Family Sylviidae (warblers). In; Hoyo JD , Elliott A, Christie DA, editors. Handbook of the Birds of the World, 11. Barcelona: Lynx Edicions; 2006. pp 492–709.
3. Collar NJ, Robson C. Family Timaliidae (babblers). In: Hoyo JD, Elliott A, Christie DA, editors. Handbook of the birds of the world, 12. Barcelona;Lynx Edicions; 2007. pp 165–171.
4. Wells DR. Family Pycnonotidae (bulbuls). In: Hoyo JD, Elliott A, Christie DA, editors. Handbook of the birds of the world, 10. Barcelona: Lynx Edicions; 2005. pp 169.

**Table S2** Summary of key statistics for sequences of each studied species*.*

| Species | Locus | Length | *N*_ind_total_ | *N*_ind_phased_ | *S*^#^ | *N*_hap_^#^ | *π*^#^ | Tajima’s D^#^ | Fu and Li’ D*^#^ |
| --- | --- | --- | --- | --- | --- | --- | --- | --- | --- |
| *Aegithalos concinnus* | BEGAIN | 255 | 29 | 29 | 6 | 7 | 0.00218 | 1.4120 (0.04050) | -0.6006 (0.23910) |
|  | AMER3 | 425 | 29 | 27 | 12 | 14 | 0.00395 | -1.0568 (0.14830) | -1.8266 (0.06750) |
|  | ZNF618 | 390 | 29 | 27 | 7 | 9 | 0.00285 | -0.7260 (0.27280) | 0.4465 (0.57520) |
|  | RNF6 | 326 | 28 | 26 | 7 | 8 | 0.00298 | -0.9779 (0.18580) | -1.1145 (-0.15650) |
|  | JMJD1C | 404 | 29 | 27 | 8 | 11 | 0.00276 | -0.9809 (0.18030) | -0.1531 (0.38810) |
|  | GPRC6A | 603 | 29 | 27 | 15 | 14 | 0.00384 | -0.8984 (0.20250) | 0.6357 (0.73330) |
|  | TET1 | 423 | 29 | 27 | 7 | 8 | 0.00255 | -0.7734 (0.25370) | 0.4465 (0.58540) |
|  | TMEM132B | 335 | 29 | 26 | 5 | 6 | 0.00218 | -0.8162 (0.24750) | -0.8449 (0.24870) |
|  | ZBTB43 | 480 | 29 | 29 | 4 | 7 | 0.00229 | 0.5981 (0.75010) | -0.1359 (0.43330) |
|  | GPR15 | 582 | 28 | 28 | 3 | 4 | 0.00041 | -1.2832 (0.03090) | -1.7671 (0.09440) |
|  | CCR7 | 376 | 27 | 25 | 8 | 10 | 0.00274 | -1.1548 (0.12200) | -1.1073 (0.16170) |
|  | SHANK2 | 320 | 29 | 26 | 7 | 8 | 0.00292 | -1.0419 (0.16780) | -0.3299 (0.35550) |
|  | PHLPP2 | 620 | 29 | 29 | 3 | 4 | 0.00027 | -1.4783 (0.00200)^$^ | -1.7821 (0.08740) |
|  | HERC1 | 686 | 28 | 28 | 1 | 2 | 0.00005 | -1.0912 (0.13470) | -1.8739 (0.04420) ^$^ |
|  | TTC28 | 367 | 29 | 27 | 11 | 14 | 0.00380 | -1.2145 (0.10390) | -0.9013 (0.18680) |
|  | KIAA1462 | 386 | 29 | 25 | 7 | 12 | 0.00524 | 0.7789 (0.81210) | 1.2417 (0.93630) |
|  | RBM6 | 346 | 28 | 28 | 6 | 8 | 0.00186 | -1.2629 (0.07540) | -2.3408 (0.02980) |
|  | P2RY8 | 532 | 29 | 27 | 14 | 13 | 0.00383 | -1.0081 (0.16260) | -0.9229 (0.16670) |
|  | CCR4 | 595 | 29 | 28 | 6 | 8 | 0.00148 | -0.8162 (0.24850) | 0.2892 (0.55690) |
|  | LOC100225317 | 339 | 29 | 26 | 11 | 12 | 0.00401 | -1.2774 (0.09130) | -1.4602 (0.09110) |
|  | USP38 | 418 | 28 | 28 | 2 | 3 | 0.00138 | 0.5633 (0.75590) | 0.7360 (0.66740) |
|  | DACT2 | 296 | 26 | 23 | 8 | 9 | 0.00341 | -1.2334 (0.10570) | -0.7987 (0.22360) |
|  | KLF15 | 474 | 29 | 27 | 11 | 15 | 0.00315 | -1.0939 (0.15010) | -0.6799 (0.25300) |
|  | UBN1 | 486 | 28 | 25 | 12 | 12 | 0.00251 | -1.6121 (0.02510) ^$^ | -1.7651 (0.06890) |
|  | CEP350 | 399 | 28 | 28 | 2 | 3 | 0.00035 | -1.1824 (0.01780) ^$^ | -0.9243 (0.11910) |
| *Alcippe morrisonia* | BEGAIN | 476 | 31 | 30 | 5 | 6 | 0.00193 | -0.3357 (0.41800) | 1.0807 (0.93520) |
|  | AMER3 | 570 | 28 | 27 | 10 | 10 | 0.00330 | -0.4008 (0.38860) | 0.7718 (0.78510) |
|  | ZNF618 | 411 | 31 | 31 | 1 | 2 | 0.00015 | -0.8939 (0.19920) | 0.5256 (0.70600) |
|  | RNF6 | 498 | 31 | 30 | 8 | 9 | 0.00213 | -1.2189 (0.09300) | -0.7107 (0.24500) |
|  | LOC100217958 | 574 | 30 | 30 | 4 | 5 | 0.00136 | -0.1974 (0.44805) | 0.9869 (0.85750) |
|  | GAPDH | 336 | 28 | 28 | 7 | 13 | 0.00790 | 1.9208 (0.97240) | 1.2343 (0.92440) |
|  | RAG1 | 787 | 30 | 30 | 5 | 8 | 0.00090 | -0.7898 (0.24690) | 1.0807 (0.96400) |
|  | Neural-cadherin-like | 429 | 29 | 29 | 11 | 14 | 0.00823 | 1.3785 (0.92580) | 1.4379 (0.96920) |
|  | RPRD2 | 411 | 30 | 28 | 7 | 8 | 0.00303 | -0.4763 (0.36640) | 0.4388 (0.59360) |
|  | TET1 | 512 | 25 | 25 | 2 | 3 | 0.00061 | -0.5283 (0.28520) | -0.8929 (0.20830) |
|  | TMEM132B | 329 | 31 | 30 | 5 | 6 | 0.00265 | -0.4366 (0.37760) | 1.0807 (0.94470) |
|  | ZBTB43 | 729 | 31 | 31 | 4 | 6 | 0.00059 | -1.0806 (0.08920) | 0.9838 (0.97780) |
|  | GPR15 | 599 | 31 | 27 | 8 | 10 | 0.00278 | -0.1405 (0.50130) | 0.5716 (0.67060) |
|  | CCR7 | 350 | 26 | 25 | 12 | 15 | 0.00841 | 0.2903 (0.66350) | 1.4773 (0.97230) |
|  | SHANK2 | 647 | 31 | 29 | 13 | 13 | 0.00311 | -0.8275 (0.22440) | 0.4542 (0.66730) |
|  | BCR | 518 | 30 | 30 | 7 | 8 | 0.00169 | -1.0658 (0.16370) | 0.4242 (0.55150) |
|  | HERC1 | 630 | 30 | 30 | 3 | 4 | 0.00048 | -1.0537 (0.14810) | -1.7964 (0.10640) |
|  | TTC28 | 400 | 30 | 29 | 8 | 8 | 0.00283 | -0.9179 (0.20510) | -0.9124 (0.19220) |
|  | KIAA1462 | 683 | 29 | 28 | 7 | 7 | 0.00132 | -0.0554 (0.16420) | 0.4388 (0.55460) |
|  | RBM6 | 646 | 31 | 29 | 10 | 12 | 0.00112 | -0.1349 (0.50400) | -0.5086 (0.29080) |
|  | FIB | 413 | 30 | 28 | 8 | 6 | 0.00400 | -0.1404 (0.50880) | -0.8955 (0.20250) |
|  | PES1 | 493 | 29 | 26 | 10 | 12 | 0.00270 | -1.0899 (0.14510) | 0.7780 (0.76480) |
|  | Am83 | 310 | 28 | 27 | 6 | 8 | 0.00213 | -1.2470 (0.09600) | 0.2975 (0.49300) |
|  | HMGB2 | 422 | 30 | 28 | 6 | 10 | 0.00227 | -0.6701 (0.31300) | -0.5874 (0.26000) |
|  | Am39 | 507 | 27 | 27 | 8 | 9 | 0.00133 | -1.2367 (0.10200) | -0.4431 (0.29500) |
| *Cettia fortipes* | BEGAIN | 412 | 27 | 27 | 6 | 7 | 0.00127 | -1.5078 (0.02000)^$^ | -1.4448 (0.10100) |
|  | ZNF618 | 468 | 23 | 23 | 3 | 4 | 0.00045 | -1.4537 (0.00800) ^$^ | -1.6805 (0.12000) |
|  | RNF6 | 502 | 25 | 25 | 12 | 18 | 0.00410 | -0.6848 (0.29100) | 1.4773 (0.97900) |
|  | LOC100217958 | 502 | 27 | 27 | 5 | 7 | 0.00092 | -1.3841 (0.03600) ^$^ | 0.11500 (0.48900) |
|  | GPRC6A | 513 | 28 | 26 | 4 | 6 | 0.00181 | 0.1155 (0.58500) | 1.0006 (0.89700) |
|  | Neural-cadherin-like | 473 | 27 | 26 | 12 | 18 | 0.00369 | -1.0072 (0.17400 | -0.7025 (0.25800 |
|  | TET1 | 530 | 28 | 27 | 6 | 7 | 0.00128 | -1.2177 (0.08900) | -1.4448 (0.10300) |
|  | TMEM132B | 434 | 28 | 26 | 8 | 13 | 0.00236 | -1.3521 (0.07700) | -0.6446 (0.24000) |
|  | ZBTB43 | 752 | 28 | 28 | 2 | 3 | 0.00014 | -1.3144 (0.01100) ^$^ | -0.9243 (0.08400) |
|  | CCR7 | 554 | 27 | 27 | 4 | 5 | 0.00085 | -1.0341 (0.19200) | -1.2325 (0.14300) |
|  | SHANK2 | 521 | 27 | 26 | 9 | 10 | 0.00186 | -1.4289 (0.05900) | 0.0204 (0.48300) |
|  | BCR | 502 | 28 | 27 | 5 | 8 | 0.00186 | -0.7321 (0.27000) | 0.2975 (0.51000) |
|  | HERC1 | 689 | 28 | 28 | 3 | 4 | 0.00034 | -1.2832 (0.02100) | -1.7671 (0.08500) |
|  | **TTC28** | **399** | **25** | **24** | **8** | **9** | **0.00127** | **-1.9567(0.00000) ^$^** | **-2.9806 (0.00100) ^$^** |
|  | KIAA1462 | 612 | 28 | 27 | 14 | 15 | 0.00227 | -1.6425 (0.02300) ^$^ | -0.4293 (0.34600) |
|  | RBM6 | 571 | 27 | 26 | 4 | 6 | 0.00088 | -0.9693 (0.20000) | 1.0006 (0.97300) |
|  | P2RY8 | 510 | 28 | 27 | 7 | 7 | 0.00155 | -1.2691 (0.08800) | -1.1338 (0.14900) |
|  | CCR4 | 634 | 25 | 25 | 3 | 3 | 0.00031 | -1.4593 (0.01500) ^$^ | -0.41300 (0.17900) |
|  | LOC100225317 | 409 | 25 | 25 | 5 | 8 | 0.00215 | -0.5112 (0.34400) | 0.1339 (0.46800) |
|  | SETD1B | 527 | 26 | 26 | 5 | 7 | 0.00157 | -0.6049 (0.31600) | 1.0933 (0.95200) |
|  | USP38 | 418 | 28 | 28 | 7 | 10 | 0.00195 | -1.2065 (0.10400) | 0.4388 (0.53800) |
|  | DIDO1 | 458 | 28 | 28 | 6 | 8 | 0.00260 | -0.2197 (0.44900) | 0.2892 (0.61600) |
|  | KLF15 | 580 | 28 | 27 | 4 | 5 | 0.00112 | -0.5794 (0.32200) | 0.9969 (0.90000) |
|  | CEP350 | 411 | 27 | 27 | 8 | 10 | 0.00275 | -0.9563 (0.20900) | -1.6026 (0.08800) |
|  | ODZ1 | 562 | 28 | 28 | 3 | 4 | 0.00069 | -0.8166 (0.25100) | -0.4435 (0.27100) |
|  | LOC100229969 | 449 | 25 | 24 | 10 | 12 | 0.00218 | -1.6284 (0.01700) | -1.0328 (0.16400) |
| *Fulvetta ruficapilla* | BEGAIN | 691 | 17 | 17 | 2 | 3 | 0.00033 | -1.0535 (0.12700) | 0.7903 (0.97500) |
|  | AMER3 | 468 | 17 | 15 | 4 | 5 | 0.00084 | -1.5738 (0.00710)^$^ | -0.9679 (0.14600) |
|  | ZNF618 | 669 | 16 | 16 | 2 | 3 | 0.00027 | -1.2671 (0.02700) ^$^ | -0.7585 (0.15200) |
|  | RNF6 | 804 | 16 | 16 | 4 | 8 | 0.00146 | 0.4629 (0.69900) | 1.0508 (0.84300) |
|  | JMJD1C | 623 | 17 | 17 | 2 | 3 | 0.00069 | -0.2404 (0.35000) | -0.7779 (0.23200) |
|  | **Neural-cadherin-like** | **631** | **17** | **-** | **0** | **1** | **0** | **-** |  |
|  | TET1 | 702 | 17 | 17 | 2 | 3 | 0.00032 | -1.0679 (0.12900) | -0.7779 (0.20300) |
|  | ZBTB43 | 745 | 17 | 17 | 2 | 3 | 0.00073 | 0.2273 (0.65700) | -0.7779 (0.22500) |
|  | GPR15 | 621 | 17 | 17 | 2 | 3 | 0.00045 | -0.8448 (0.19300) | 0.7903 (0.95300) |
|  | CCR7 | 668 | 17 | 17 | 5 | 6 | 0.00076 | -1.5456 (0.01600) ^$^ | -1.5634 (0.12100) |
|  | SHANK2 | 431 | 17 | 17 | 4 | 5 | 0.00287 | 0.6574 (0.76500) | 1.0442 (0.84000) |
|  | PHLPP2 | 634 | 17 | 17 | 2 | 3 | 0.00027 | -1.2766 (0.02010) ^$^ | -0.7779 (0.14950) |
|  | BCR | 594 | 17 | 17 | 8 | 4 | 0.00444 | 1.0287 (0.86100) | 1.3184 (0.95100) |
|  | HERC1 | 694 | 17 | 17 | 2 | 3 | 0.00039 | -0.8736 (0.17500) | -0.7779 (0.17000) |
|  | TTC28 | 537 | 17 | 17 | 5 | 7 | 0.00312 | 0.3961 (0.71500) | 0.4078 (0.59100) |
|  | KIAA1462 | 719 | 17 | 17 | 2 | 3 | 0.00024 | -1.2766 (0.02200) ^$^ | -0.7779 (0.15800) |
|  | RBM6 | 675 | 17 | 17 | 3 | 5 | 0.00132 | 0.4772 (0.73100) | -0.3017 (0.39300) |
|  | P2RY8 | 765 | 17 | 17 | 3 | 4 | 0.00058 | -0.9037 (0.19500) | -0.9345 (0.96800) |
|  | GPR125 | 676 | 17 | 17 | 1 | 2 | 0.00017 | -0.7993 (0.11600) | 0.5804 (0.95200) |
|  | CCR4 | 635 | 17 | 17 | 1 | 2 | 0.00009 | -1.1378 (0.12640) | -1.7229 (0.05860) |
|  | LOC100225317 | 629 | 17 | 17 | 4 | 5 | 0.00071 | -1.3530 (0.04600) | -1.0277 (0.13300) |
|  | SETD1B | 339 | 17 | 17 | 1 | 2 | 0.00126 | 1.1225 (0.87270) | 0.5804 (0.75360) |
|  | **USP38** | **656** | **17** | **-** | **1** | **0** | **0** | **-** | **-** |
|  | TYR | 675 | 17 | 17 | 2 | 3 | 0.00026 | -1.2766 (0.01600) ^$^ | -0.7779 (0.15000) |
|  | DACT2 | 699 | 17 | 17 | 4 | 5 | 0.00127 | -0.2256 (0.43550) | 0.0082 (0.42400) |
|  | KLF15 | 664 | 17 | 17 | 1 | 2 | 0.00025 | -0.4827 (0.20900) | 0.5804 (0.91790) |
|  | CEP350 | 630 | 17 | 17 | 1 | 2 | 0.00064 | 0.9587 (0.83840) | 0.5804 (0.76640) |
| *Leiothrix lutea* | **ZNF618** | **477** | **25** | **25** | **8** | **10** | **0.00121** | **-1.8470 (0.00000)^$^** | **-2.2671(0.03800) ^$^** |
|  | RNF6 | 462 | 26 | 25 | 9 | 21 | 0.00424 | -0.3512 (0.41000) | 0.7845 (0.74800) |
|  | LOC100217958 | 507 | 26 | 26 | 10 | 12 | 0.00165 | -1.7643 (0.00800) | -1.0798 (0.15100) |
|  | JMJD1C | 401 | 26 | 24 | 5 | 7 | 0.00166 | -0.9961 (0.19400) | 1.1004 (0.96800) |
|  | GPRC6A | 298 | 26 | 26 | 6 | 8 | 0.00425 | -0.1143 (0.50300) | 0.3062 (0.54600) |
|  | Neural-cadherin-like | 373 | 26 | 23 | 10 | 9 | 0.00305 | -1.4499 (0.05700) | -1.0077 (0.16500) |
|  | TET1 | 419 | 25 | 22 | 8 | 10 | 0.00254 | -1.1799 (0.12800) | -0.7764 (0.21300) |
|  | TMEM132B | 294 | 24 | 23 | 8 | 9 | 0.00337 | -1.2643 (0.09600) | -0.7987 (0.19700) |
|  | ZBTB43 | 760 | 26 | 25 | 3 | 4 | 0.00026 | -1.4643 (0.00800) ^$^ | -1.7178 (0.10700) |
|  | GPR15 | 526 | 26 | 26 | 9 | 7 | 0.00497 | 0.8671 (0.82300) | 0.6855 (0.75700) |
|  | CCR7 | 349 | 25 | 24 | 6 | 8 | 0.00259 | -0.8501 (0.24140) | -0.5283 (0.27560) |
|  | SHANK2 | 374 | 26 | 22 | 10 | 13 | 0.00432 | -1.0741 (0.14730) | -1.3431 (0.10240) |
|  | PHLPP2 | 610 | 25 | 25 | 1 | 2 | 0.00054 | 0.6215 (0.80480) | 0.5432 (0.77860) |
|  | **BCR** | **497** | **25** | **22** | **12** | **12** | **0.00127** | **-2.3325 (0.00000) ^$^** | **-3.7537 (0.00020) ^$^** |
|  | HERC1 | 686 | 26 | 26 | 8 | 9 | 0.00096 | -1.7002 (0.00560) ^$^ | -1.5786 (0.10880) |
|  | TTC28 | 252 | 25 | 25 | 7 | 9 | 0.00393 | -0.9693 (0.18530) | -1.0943 (0.14980) |
|  | KIAA1462 | 521 | 26 | 25 | 13 | 15 | 0.00478 | -0.4236 (0.37770) | 1.5115 (0.98090) |
|  | RBM6 | 610 | 25 | 25 | 12 | 13 | 0.00147 | -1.9690 (0.00160) ^$^ | -0.6843 (0.24460) |
|  | P2RY8 | 566 | 26 | 26 | 9 | 9 | 0.00131 | -1.7414 (0.00700) ^$^ | -0.6446 (0.25260) |
|  | GPR125 | 434 | 25 | 22 | 7 | 9 | 0.00451 | 0.5843 (0.75130) | 0.4908 (0.67840) |
|  | ARSI | 281 | 24 | 22 | 9 | 12 | 0.00438 | -1.1637 (0.11480) | -1.2065 (0.12260) |
|  | CCR4 | 390 | 26 | 25 | 6 | 6 | 0.00151 | -1.4288 (0.04650) ^$^ | -1.4035 (0.11880) |
|  | SETD1B | 484 | 26 | 22 | 16 | 24 | 0.00487 | -1.1368 (0.12820) | -0.5224 (0.27780) |
|  | USP38 | 619 | 26 | 24 | 9 | 10 | 0.00169 | -1.3668 (0.06650) | 0.0465 (0.48640) |
|  | KLF15 | 508 | 25 | 23 | 11 | 12 | 0.00262 | -1.3812 (0.06860) | -1.3745 (0.09470) |
|  | UBN1 | 384 | 26 | 25 | 9 | 11 | 0.00276 | -1.3207 (0.07240) | -0.6263 (0.24650) |
|  | CEP350 | 261 | 26 | 20 | 10 | 14 | 0.00547 | -1.1723 (0.11590) | -1.5057 (0.10700) |
| *Lioparus chrysotis* | BEGAIN | 581 | 22 | 22 | 6 | 9 | 0.00259 | 0.2347 (0.64010) | 1.1846 (0.90140) |
|  | AMER3 | 422 | 25 | 25 | 7 | 10 | 0.00355 | -0.1111 (050370) | 1.2417 (0.94730) |
|  | ZNF618 | 641 | 22 | 22 | 3 | 4 | 0.00042 | -1.3069 (0.02890)^$^ | 0.9052 (0.98530) |
|  | RNF6 | 809 | 23 | 23 | 2 | 3 | 0.00038 | -0.5916 (0.27480) | -0.8691 (0.19580) |
|  | JMJD1C | 446 | 25 | 24 | 5 | 8 | 0.00342 | 0.8639 (0.83150) | 0.1440 (0.53990) |
|  | Neural-cadherin-like | 561 | 25 | 25 | 5 | 6 | 0.00172 | -0.3233 (0.40890) | 1.0968 (0.93280) |
|  | TET1 | 366 | 25 | 22 | 5 | 7 | 0.00248 | -0.5277 (0.34000) | -0.7763 (0.25540) |
|  | ZBTB43 | 724 | 23 | 23 | 2 | 3 | 0.00029 | -0.9821 (0.13170) | 0.7560 (0.96920) |
|  | GPR15 | 545 | 23 | 22 | 6 | 8 | 0.00221 | -0.3342 (0.41270) | 1.1846 (0.91800) |
|  | CCR7 | 552 | 25 | 23 | 6 | 7 | 0.00234 | -0.1356 (0.49300) | -0.5116 (0.29190) |
|  | SHANK2 | 374 | 24 | 24 | 5 | 9 | 0.00407 | 0.8543 (0.82610) | 0.1440 (0.53660) |
|  | PHLPP2 | 635 | 24 | 24 | 1 | 2 | 0.00013 | -0.8664 (0.20940) | 0.5467 (0.72100) |
|  | BCR | 467 | 24 | 23 | 5 | 7 | 0.00220 | -0.2419 (0.44920) | 0.1547 (0.48870) |
|  | HERC1 | 603 | 25 | 25 | 3 | 5 | 0.00086 | -0.4620 (0.34250) | 0.8917 (0.92930) |
|  | TTC28 | 583 | 24 | 23 | 2 | 3 | 0.00053 | -0.8691 (0.19990) | -0.5961 (0.26670) |
|  | KIAA1462 | 634 | 24 | 24 | 2 | 3 | 0.00019 | -1.3093 (0.01290) | -0.8813 (0.10540) |
|  | RBM6 | 552 | 24 | 24 | 3 | 4 | 0.00126 | 0.0560 (0.58870) | 0.8960 (0.79080) |
|  | P2RY8 | 684 | 24 | 24 | 1 | 2 | 0.00045 | 0.5173 (0.77300) | 0.5467 (0.52240) |
|  | GPR125 | 668 | 24 | 24 | 6 | 6 | 0.00152 | -0.6397 (0.29950) | 1.1778 (0.93590) |
|  | CCR4 | 635 | 25 | 25 | 3 | 4 | 0.00031 | -1.4643 (0.01260) | -1.7178 (0.09910) |
|  | LOC100225317 | 630 | 25 | 25 | 3 | 4 | 0.00089 | -0.3311 (0.39090) | -0.4130 (0.32800) |
|  | SETD1B | 483 | 24 | 24 | 5 | 6 | 0.00134 | -1.0384 (0.16830) | 1.1004 (0.97660) |
|  | USP38 | 654 | 25 | 25 | 3 | 4 | 0.00078 | -0.4973 (0.33880) | -1.7178 (0.11460) |
|  | TYR | 536 | 24 | 24 | 3 | 4 | 0.00105 | -0.3433 (0.38800) | -0.4018 (0.32480) |
|  | DACT2 | 446 | 24 | 24 | 1 | 2 | 0.00111 | 1.6340 (0.94770) | 0.5467 (0.50690) |
|  | KLF15 | 653 | 25 | 25 | 2 | 3 | 0.00018 | -1.3111 (0.00990) | -0.8929 (0.10480) |
| *Parus monticolus* | BEGAIN | 769 | 19 | 17 | 9 | 11 | 0.00194 | -0.9739 (0.17840) | -1.0420 (0.15240) |
|  | AMER3 | 470 | 19 | 19 | 7 | 7 | 0.00193 | -1.2722 (0.08940) | -0.9484 (0.17210) |
|  | ZNF618 | 722 | 19 | 19 | 3 | 4 | 0.00042 | -1.2668 (0.04080)^$^ | -0.3351 (0.24270) |
|  | **RNF6** | 782 | 19 | 18 | 3 | 4 | 0.00028 | -0.00935 (0.004) ^$^ | 0.04543 (0.10100) |
|  | LOC100217958 | **676** | **19** | **19** | **3** | **4** | **0.00023** | **-1.7200 (0.00070) ^$^** | **-2.8484 (0.00090) ^$^** |
|  | JMJD1C | 635 | 18 | 18 | 1 | 2 | 0.00009 | -1.1332 (0.12090) | -1.7411 (0.06280) |
|  | GPRC6A | 555 | 19 | 19 | 5 | 5 | 0.00185 | -0.3527 (0.40720) | 0.2039 (0.54770) |
|  | Neural-cadherin-like | 433 | 19 | 17 | 3 | 4 | 0.00152 | -0.2298 (0.42900) | -0.3017 (0.36340) |
|  | RPRD2 | 398 | 19 | 17 | 5 | 6 | 0.00201 | -0.9174 (0.19770) | -1.5634 (0.10270) |
|  | TET1 | 490 | 19 | 18 | 3 | 5 | 0.00302 | 2.3350 (0.99080) | 0.9277 (0.79790) |
|  | TMEM132B | 648 | 19 | 19 | 3 | 4 | 0.00039 | -1.4208 (0.02110) ^$^ | -1.5918 (0.11900) |
|  | ZBTB43 | 705 | 19 | 19 | 2 | 3 | 0.00051 | -0.4613 (0.30670) | -0.8125 (0.22980) |
|  | GPR15 | 636 | 19 | 19 | 3 | 4 | 0.00183 | 1.3948 (0.91650) | 0.9215 (0.77500) |
|  | CCR7 | 565 | 19 | 18 | 3 | 5 | 0.00136 | 0.1387 (0.61160) | 0.9277 (0.80420) |
|  | SHANK2 | 644 | 19 | 17 | 4 | 5 | 0.00147 | -0.0807 (0.50330) | -1.0277 (0.17700) |
|  | **PHLPP2** | **513** | **19** | **-** | **0** | **1** | **0** | **-** | **-** |
|  | BCR | 573 | 19 | 19 | 3 | 4 | 0.00053 | -1.2668 (0.04140) ^$^ | -0.7078 (0.18280) |
|  | HERC1 | 624 | 19 | 19 | 3 | 4 | 0.00101 | -0.2506 (0.42250) | -0.3351 (0.34320) |
|  | TTC28 | 548 | 19 | 19 | 2 | 3 | 0.00084 | -0.0723 (0.44450) | 0.7771 (0.73460) |
|  | RBM6 | 610 | 19 | 19 | 3 | 4 | 0.00070 | -0.8841 (0.19870) | -0.3351 (0.30070) |
|  | P2RY8 | 747 | 19 | 19 | 2 | 3 | 0.00073 | 0.2710 (0.68320) | -0.8125 (0.23880) |
|  | GPR125 | 592 | 19 | 18 | 4 | 8 | 0.00252 | 1.3354 (0.91530) | 1.0380 (0.87900) |
|  | ARSI | 558 | 19 | 18 | 4 | 13 | 0.00345 | 2.4357 (0.99420) | 1.0380 (0.87410) |
|  | CCR4 | 490 | 19 | 19 | 6 | 6 | 0.00128 | -1.4973 (0.02180) ^$^ | -1.2503 (0.13290) |
|  | LOC100225317 | 404 | 17 | 17 | 1 | 2 | 0.00015 | -1.1378 (0.11920) | -1.7229 (0.05790) |
|  | SETD1B | 782 | 19 | 18 | 4 | 6 | 0.00132 | 0.1745 (0.61920) | -0.0082 (0.47390) |
|  | USP38 | 615 | 19 | 19 | 3 | 4 | 0.00113 | -0.0570 (0.48550) | 0.9215 (0.81060) |
|  | TYR | 481 | 17 | 17 | 3 | 4 | 0.00093 | -0.8927 (0.18470) | 0.9345 (0.95850) |
| *Pomatorhinus ruficollis* | BEGAIN | 365 | 34 | 32 | 9 | 11 | 0.00243 | -1.4134 (0.05100) | -0.7539 (0.22960) |
|  | ZNF618 | 674 | 33 | 33 | 3 | 4 | 0.00091 | -0.4509 (0.34970) | -1.8483 (0.10060) |
|  | RNF6 | 504 | 34 | 33 | 7 | 8 | 0.00160 | -1.1386 (0.11960) | -1.2340 (0.13360) |
|  | LOC100217958 | 534 | 34 | 33 | 6 | 8 | 0.00202 | -0.3482 (0.42520) | 0.2523 (0.56940) |
|  | JMJD1C | 374 | 34 | 32 | 9 | 10 | 0.00350 | -0.8359 (0.23310) | 0.6479 (0.69480) |
|  | GPRC6A | 401 | 34 | 32 | 8 | 11 | 0.00390 | -0.1965 (0.48270) | -0.2105 (0.38870) |
|  | GAPDH | 441 | 34 | 32 | 3 | 4 | 0.00055 | -0.1976 (0.43880) | -1.8234 (0.09990) |
|  | Neural-cadherin-like | 291 | 34 | 31 | 8 | 11 | 0.00433 | -0.9216 (0.20200) | 0.6537 (0.69620) |
|  | RPRD2 | 645 | 33 | 33 | 4 | 5 | 0.00111 | -0.3098 (0.42090) | -0.1680 (0.4107) |
|  | TET1 | 655 | 34 | 32 | 7 | 8 | 0.00111 | -1.2869 (0.08030) | -1.2189 (0.13390) |
|  | TMEM132B | 455 | 34 | 32 | 5 | 6 | 0.00165 | -0.6641 (0.29860) | 0.0743 (0.49800) |
|  | ZBTB43 | 687 | 34 | 34 | 2 | 3 | 0.00084 | 0.6304 (0.77430) | 0.7178 (0.65380) |
|  | GPR15 | 518 | 34 | 34 | 5 | 6 | 0.00096 | -1.1917 (0.08350) | 0.0601 (0.45760) |
|  | CCR7 | 500 | 34 | 34 | 6 | 7 | 0.00200 | -0.4826 (0.35900) | -0.6593 (0.25470) |
|  | Am44 | 426 | 32 | 31 | 14 | 17 | 0.00617 | -0.3479 (0.41800) | 0.5262 (0.67380) |
|  | **SHANK2** | **386** | **20** | **20** | **4** | **4** | **0.00064** | **-1.7592(0.00040) ^$^** | **-2.1673(0.0159) ^$^** |
|  | PHLPP2 | 534 | 34 | 34 | 1 | 2 | 0.00011 | -0.9002 (0.19370) | 0.5186 (0.70560) |
|  | BCR | 393 | 32 | 31 | 7 | 9 | 0.00258 | -0.8109 (0.24920) | -0.3930 (0.35270) |
|  | **TTC28** | **556** | **34** | **31** | **12** | **11** | **0.00108** | **-2.1816 (0.00010) ^$^** | **-3.0728 (0.00500) ^$^** |
|  | KIAA1462 | 446 | 30 | 29 | 6 | 7 | 0.00316 | 0.2163 (0.64470) | -0.6006 (0.27160) |
|  | AM102 | 364 | 34 | 29 | 5 | 7 | 0.00270 | -0.210 (0.47320) | -0.8883 (0.23570) |
|  | Sr24 | 496 | 33 | 30 | 9 | 9 | 0.00206 | -1.2576 (0.09450) | -0.0360 (0.43730) |
|  | GIF2B | 302 | 34 | 32 | 7 | 7 | 0.00378 | -0.5760 (0.32500) | 1.2253 (0.94090) |
|  | **Am47** | **309** | **18** | **17** | **5** | **5** | **0.0016** | **-1.5764(0.01070) ^$^** | **-2.4618(0.00710) ^$^** |
|  | Am83 | 353 | 34 | 31 | 20 | 17 | 0.00930 | -0.7070 (0.25800) | 0.5238 (0.66600) |
|  | ODC | 479 | 30 | 30 | 10 | 11 | 0.00248 | -1.2206 (0.10500) | -0.5377 (0.22700) |
|  | **myo** | **522** | **18** | **18** | **0** | **1** | **0** | **-** | **-** |
|  | HMGB2 | 334 | 31 | 28 | 6 | 9 | 0.00418 | 0.1702 (0.63400) | 0.2892 (0.59800) |
|  | CCDC132 | 308 | 31 | 29 | 7 | 9 | 0.00303 | -0.9497 (0.19800) | -1.2032 (0.14700) |
| *Spizixos semitorques* | BEGAIN | 387 | 17 | 17 | 4 | 5 | 0.00104 | -1.4662 (0.04000)^$^ | 0.00082 (0.48700) |
|  | AMER3 | 516 | 18 | 28 | 5 | 6 | 0.00132 | -1.1316 (0.14400) | -0.6902 (0.25500) |
|  | ZNF618 | 444 | 18 | 17 | 5 | 6 | 0.00097 | -1.6741 (0.00650) ^$^ | -1.5986 (0.11840) |
|  | RNF6 | 585 | 18 | 18 | 4 | 5 | 0.00142 | -0.3414 (0.38200) | 1.0380 (0.94200) |
|  | LOC100217958 | 631 | 18 | 18 | 2 | 3 | 0.00041 | -0.9007 (0.15370) | -0.7958 (0.19220) |
|  | JMJD1C | 332 | 18 | 17 | 6 | 8 | 0.00484 | 0.2650 (0.64700) | 0.4078 (0.60200) |
|  | GPRC6A | 553 | 18 | 17 | 6 | 6 | 0.00333 | 0.7089 (0.78970) | 0.4078 (0.61520) |
|  | Neural-cadherin-like | 407 | 18 | 18 | 1 | 2 | 0.00060 | 0.0298 (0.72980) | 0.4860 (0.71610) |
|  | TET1 | 530 | 18 | 18 | 4 | 5 | 0.00138 | -0.5913 (0.30900) | -0.0082 (0.43480) |
|  | TMEM132B | 376 | 17 | 17 | 3 | 4 | 0.00100 | -1.1081 (0.14800) | -1.5379 (0.13100) |
|  | GPR15 | 431 | 18 | 18 | 6 | 5 | 0.00231 | -0.8499 (0.22140) | -0.4127 (0.30170) |
|  | CCR7 | 490 | 18 | 18 | 3 | 4 | 0.00099 | -0.7359 (0.25840) | -0.3190 (0.25560) |
|  | SHANK2 | 293 | 18 | 18 | 7 | 9 | 0.00524 | -0.2543 (0.45150) | -0.1917 (0.40580) |
|  | HERC1 | 406 | 18 | 18 | 3 | 4 | 0.00135 | -0.5443 (0.29850) | -0.3190 (0.26930) |
|  | TTC28 | 360 | 18 | 18 | 4 | 6 | 0.00274 | 0.0576 (0.57710) | 1.0380 (0.91140) |
|  | KIAA1462 | 536 | 18 | 18 | 2 | 3 | 0.00031 | -1.2844 (0.01380) ^$^ | -0.7958 (0.13670) |
|  | RBM6 | 382 | 18 | 17 | 5 | 8 | 0.00378 | 0.4740 (0.71500) | 1.1320 (0.91160) |
|  | P2RY8 | 503 | 18 | 18 | 2 | 3 | 0.00156 | 0.8898 (0.82040) | 0.7834 (0.68220) |
|  | GPR125 | 454 | 18 | 18 | 2 | 3 | 0.00069 | -0.6769 (0.24790) | 0.4210 (0.66730) |
|  | ARSI | 461 | 18 | 17 | 8 | 10 | 0.00347 | -0.5371 (0.32900) | 0.6644 (0.70230) |
|  | CCR4 | 654 | 18 | 18 | 6 | 9 | 0.00177 | -0.5442 (0.34340) | 0.1255 (0.51160) |
|  | LOC100225317 | 579 | 18 | 27 | 3 | 4 | 0.00109 | -0.3237 (0.38300) | 0.9345 (0.91470) |
|  | SETD1B | 700 | 18 | 17 | 3 | 4 | 0.00071 | -0.7435 (0.25810) | 0.9345 (0.94390) |
|  | USP38 | 665 | 17 | 17 | 2 | 3 | 0.00026 | -1.2766 (0.02120) ^$^ | -0.7779 (0.15100) |
|  | TYR | 591 | 18 | 18 | 5 | 5 | 0.00132 | -0.9154 (0.19960) | -1.5986 (0.10020) |
|  | DACT2 | 499 | 18 | 18 | 3 | 4 | 0.00084 | -0.9423 (0.17670) | 0.9277 (0.89650) |
| *Stachyridopsis ruficeps* | BEGAIN | 499 | 30 | 28 | 11 | 14 | 0.00394 | -0.4706 (0.36500) | 0.2390 (0.56000) |
|  | AMER3 | 424 | 31 | 27 | 2 | 4 | 0.00224 | 2.0356 (0.97660) | 0.7396 (0.67560) |
|  | ZNF618 | 525 | 31 | 31 | 6 | 5 | 0.00137 | -1.0711 (0.16160) | -0.6255 (0.25720) |
|  | RNF6 | 532 | 31 | 27 | 8 | 11 | 0.00200 | -1.0505 (0.16610) | 1.2963 (0.97260) |
|  | LOC100217958 | 530 | 31 | 31 | 4 | 4 | 0.00081 | -1.0699 (0.16050) | 0.9838 (0.97600) |
|  | JMJD1C | 538 | 31 | 29 | 6 | 8 | 0.00202 | -0.4034 (0.39210) | 0.2812 (0.56590) |
|  | GPRC6A | 528 | 31 | 31 | 6 | 9 | 0.00189 | -0.5345 (0.34300) | -0.6255 (0.25700) |
|  | Neural-cadherin-like | 269 | 31 | 30 | 7 | 7 | 0.00399 | -0.7301 (0.25800) | 1.2297 (0.94700) |
|  | RPRD2 | 549 | 30 | 28 | 6 | 6 | 0.00095 | -1.4981 (0.01400)^$^ | 1.1659 (0.99400) |
|  | TET1 | 490 | 31 | 30 | 6 | 9 | 0.00272 | 0.0859 (0.59740) | 1.1605 (0.90050) |
|  | TMEM132B | 424 | 31 | 31 | 8 | 10 | 0.00208 | -1.2674 (0.10800) | -0.9443 (0.21200) |
|  | ZBTB43 | 750 | 31 | 30 | 1 | 2 | 0.00051 | 0.9989 (0.83400) | 0.5281 (0.50300) |
|  | GPR15 | 451 | 31 | 29 | 4 | 5 | 0.00204 | 0.1379 (0.57900) | -0.1359 (0.43100) |
|  | SHANK2 | 526 | 31 | 30 | 11 | 13 | 0.00389 | -0.7755 (0.22600) | -0.0881 (0.44700) |
|  | **PHLPP2** | **631** | **31** | **0** | **0** | **1** | **0** | **-** | **-** |
|  | BCR | 377 | 31 | 31 | 5 | 7 | 0.00174 | -0.8936 (0.24200) | 0.0818 (0.50500) |
|  | HERC1 | 614 | 31 | 31 | 5 | 5 | 0.00086 | -1.1702 (0.07300) | 0.0818 (0.45200) |
|  | TTC28 | 483 | 31 | 31 | 7 | 9 | 0.00228 | -0.6630 (0.29200) | -0.3930 (0.32800) |
|  | KIAA1462 | 753 | 31 | 31 | 11 | 13 | 0.00141 | -1.5317 (0.03700) ^$^ | -1.5807 (0.09100) |
|  | RBM6 | 511 | 29 | 29 | 5 | 6 | 0.00105 | -1.18836 (0.07300) | -0.8883 (0.23100) |
|  | P2RY8 | 601 | 31 | 31 | 4 | 7 | 0.00224 | 0.6117 (0.76700) | -0.9142 (0.21100) |
|  | GPR125 | 493 | 31 | 31 | 5 | 8 | 0.00227 | 0.1230 (0.58600) | 0.0818 (0.53200) |
|  | ARSI | 580 | 28 | 27 | 10 | 14 | 0.00180 | -1.4750 (0.04100) ^$^ | -0.4772 (0.27000) |
|  | CCR4 | 559 | 31 | 29 | 8 | 13 | 0.00421 | 0.5699 (0.75200) | 0.6657 (0.71900) |
|  | LOC100225317 | 480 | 29 | 29 | 7 | 11 | 0.00346 | 0.2514 (0.64500) | 1.2320 (0.90300) |
|  | SETD1B | 605 | 30 | 29 | 10 | 11 | 0.00220 | -1.0725 (0.15600) | -1.7772 (0.08200) |
|  | USP38 | 372 | 30 | 29 | 7 | 8 | 0.00431 | 0.1557 (0.61800) | 1.2320 (0.92500) |
|  | TYR | 438 | 31 | 30 | 5 | 6 | 0.00100 | -1.3804 (0.03500) ^$^ | -0.9015 (0.22800) |
|  | DIDO1 | 651 | 30 | 29 | 5 | 7 | 0.00133 | -0.4681 (0.35000) | 1.0837 (0.89400) |
|  | KLF15 | 615 | 31 | 31 | 8 | 12 | 0.00310 | 0.3117 (0.65700) | 0.5444 (0.67300) |
|  | UBN1 | 419 | 31 | 30 | 6 | 8 | 0.00213 | -0.7501 (0.27400) | -0.6133 (0.25800) |
| *Yuhina diademata* | BEGAIN | 697 | 32 | 32 | 5 | 8 | 0.00159 | 0.1105 (0.59900) | 0.0743 (0.50860) |
|  | ZNF618 | 620 | 31 | 31 | 5 | 6 | 0.00100 | -0.9708 (0.17950) | -0.2956 (0.33030) |
|  | RNF6 | 832 | 31 | 31 | 5 | 7 | 0.00086 | -0.7691 (0.25350) | 1.0778 (0.92740) |
|  | JMJD1C | 652 | 31 | 31 | 6 | 8 | 0.00063 | -1.8438 (0.00040)^$^ | -1.2032 (0.11740) |
|  | GPRC6A | 630 | 30 | 30 | 2 | 3 | 0.00062 | -0.1444 (0.42260) | 0.7294 (0.87390) |
|  | Neural-cadherin-like | 555 | 32 | 30 | 6 | 8 | 0.00288 | 0.6004 (0.76570) | 1.1521 (0.88760) |
|  | TET1 | 590 | 31 | 31 | 2 | 3 | 0.00044 | -0.6580 (0.24620) | -0.9516 (0.17420) |
|  | TMEM132B | 533 | 31 | 30 | 11 | 16 | 0.00299 | -0.9166 (0.19780) | -0.3602 (0.35160) |
|  | ZBTB43 | 742 | 32 | 32 | 1 | 2 | 0.00042 | 0.5858 (0.79620) | 0.5231 (0.77310) |
|  | GPR15 | 648 | 32 | 32 | 3 | 4 | 0.00037 | 1.2197 (0.02990) ^$^ | -0.4782 (0.19240) |
|  | CCR7 | 613 | 31 | 29 | 6 | 11 | 0.00230 | 0.2198 (0.63770) | 0.2812 (0.60390) |
|  | SHANK2 | 389 | 31 | 29 | 5 | 5 | 0.00132 | -1.2344 (0.06780) | -1.1691 (0.14210) |
|  | PHLPP2 | 591 | 31 | 31 | 1 | 2 | 0.00043 | 0.2351 (0.72570) | 0.5256 (0.80900) |
|  | HERC1 | 680 | 30 | 30 | 1 | 2 | 0.00073 | 1.7056 (0.94690) | 0.5281 (0.49950) |
|  | TTC28 | 538 | 31 | 31 | 6 | 10 | 0.00312 | 0.7691 (0.80770) | 1.1579 (0.88890) |
|  | KIAA1462 | 720 | 31 | 30 | 1 | 2 | 0.00066 | 1.5433 (0.92480) | 0.5281 (0.49560) |
|  | RBM6 | 677 | 31 | 31 | 1 | 2 | 0.00018 | -0.5397 (0.18640) | 0.5256 (0.92420) |
|  | GPR125 | 640 | 31 | 31 | 4 | 6 | 0.00140 | 0.1190 (0.58900) | 0.9838 (0.88700) |
|  | CCR4 | 612 | 32 | 32 | 5 | 8 | 0.00185 | 0.1613 (0.62220) | 1.0750 (0.86620) |
|  | LOC100225317 | 611 | 31 | 31 | 3 | 4 | 0.00164 | 1.1223 (0.87480) | 0.8701 (0.74220) |
|  | SETD1B | 156 | 31 | 31 | 6 | 7 | 0.00117 | -0.7528 (0.27400) | 0.2662 (0.52000) |
|  | USP38 | 654 | 31 | 30 | 2 | 1 | 0.00048 | -0.4389 (0.31700) | 0.7263 (0.90820) |
|  | TYR | 635 | 32 | 31 | 1 | 2 | 0.00072 | 1.4494 (0.90930) | 0.5256 (0.69400) |
|  | DACT2 | 722 | 32 | 32 | 5 | 6 | 0.00126 | -0.3244 (0.41750) | 1.0750 (0.89160) |
|  | UBN1 | 741 | 21 | 21 | 7 | 8 | 0.00270 | 0.2103 (0.62730) | -0.0656 (0.41850) |

*Abbreviations* used: *N*_ind_total_, number of individuals sequenced successfully; *N*_ind_total_, number of individuals phased successfully; *S*, number of segregating sites; *N*_hap_, number of haplotypes; *H*_d_, haplotype diversity; *π*, nucleotide diversity; ^#^, statistics for phased genotypes; ^$^, *P* < 0.05.

**Table S3.** Estimates of model fit and the relative percentage contributions of the environmental variables to the ENM models for each species studied. Values contributing most are highlighted in bold. The training AUCs were estimated for 75% of the occurrence records for each species, while the test AUCs were gained by the other 25%. For species (^*^) with significant seasonal migration, occurrence records were collected avoiding reported dispersal times, e.g. October to April for *Cettia fortipes* and October to March for *Parus monticolus*. Models for species (^**^) with fewer occurrence records (e.g. <100) were trained with all records.

|  | No. Rec | MTSS | Bio_2 (%) | Bio_3 (%) | Bio_5 (%) | Bio_7 (%) | Bio_11 (%) | Bio_14 (%) | Bio_15 (%) | Bio_16 (%) | Bio_18 (%) | Bio_19 (%) | Training AUC | Test AUC |
| --- | --- | --- | --- | --- | --- | --- | --- | --- | --- | --- | --- | --- | --- | --- |
| *Aegithalos concinnus* | 512 | 0.219 | 2.6 (0.6) | 1 (3.6) | 8.4 (2.2) | 13.5 (18) | 20.4 (**47.7**) | 1.1 (6.2) | 1.2 (1.8) | 8.9 (2.8) | **39.7** (3.5) | 3.2 (13.6) | 0.936 | 0.904 |
| *Cettia fortipes*^*^ | 240 | 0.247 | 10.2 (8.1) | 1.7 (2.2) | 7.4 (10.5) | 20.6 (18.1) | 24.7 (**43**) | 7.9 (0.8) | 0.2 (0.9) | 0.9 (6.3) | 21.7 (5.6) | 4.6 (4.4) | 0.954 | 0.921 |
| *Leiothrix lutea*^*^ | 267 | 0.222 | 0.3 (0.7) | 2 (1) | 5.4 (8.2) | 10.9 (11) | 26.7 (**45.7**) | 1.6 (3.7) | 3.4 (7.3) | 2.3 (2.3) | **45.3** (13.1) | 2.2 (7.1) | 0.948 | 0.904 |
| *Pomtorhinus ruficollis* | 334 | 0.231 | 2.1 (7.2) | 3 (2.5) | 12 (15.8) | 12.7 (15.1) | 18.1 (**32**) | 0.2 (1.1) | 0.2 (0.4) | 1.2 (2.9) | **50.1** (22.4) | 0.3 (0.7) | 0.924 | 0.910 |
| *Stachyridopsis ruficeps* | 532 | 0.209 | 8.3 (6.5) | 1 (2.2) | 9.2 (17.3) | 7.1 (19.9) | 6.5 (**22.9**) | 0.7 (6.9) | 0.6 (3.9) | 0.7 (2.7) | **65.1** (16) | 1 (1.6) | 0.936 | 0.926 |
| *Alcippe morrisonia* | 444 | 0.269 | 14.7 (5.8) | 2.5 (2.6) | 3.6 (12.3) | 7.7 (7.1) | 5.8 (14.1) | 24 (19.1) | 1.8 (2.8) | 4.2 (**23.7**) | **34.3** (10.6) | 1.3 (2) | 0.929 | 0.919 |
| *Spizixos semtorques* | 336 | 0.231 | **44** (10) | 1.6 (6.9) | 2.2 (0.7) | 8.8 (**26**) | 11.1 (20) | 14.1 (10.6) | 0.2 (1.2) | 1 (1.2) | 15.4 (17.2) | 1.7 (6.3) | 0.958 | 0.944 |
| *Fulvetta ruficapilla*^**^ | 77 | 0.210 | 0.4 (0.1) | 0.5 (1.6) | **45.1** (24.1) | 0.5 (2.2) | 17.3 (**27.8**) | 11.9 (3.4) | 1.8 (5) | 0.5 (11.5) | 14.4 (7) | 7.5 (17.4) | 0.972 | - |
| *Lioparus chrysotis*^*^*^*^* | 99 | 0.214 | 1.2 (0.9) | 0.1 (1.2) | **37.1 (54.9)** | 10.1 (2.8) | 21.2 (20.6) | 0.8 (1.7) | 0.5 (3.2) | 2.3 (1.4) | 24.1 (10.6) | 2.7 (2.6) | 0.975 | - |
| *Parus monticolus* | 329 | 0.193 | 0.9 (2.4) | 1.7 (3) | **46.7 (71.2)** | 16.7 (2.1) | 13.5 (15.6) | 0.2 (0.6) | 0.1 (0) | 1.8 (2.1) | 16.9 (0) | 1.5 (3) | 0.966 | 0.962 |
| *Yuhina diademata* | 146 |  | 6.7 (5.1) | 2.4 (3.1) | **32.6** (20.1) | 5.8 (2.5) | 21.9 (**35.8**) | 5.6 (2.3) | 0.8 (8.7) | 1.6 (0.2) | 14.9 (1.6) | 7.6 (20.6) | 0.974 | 0.959 |

*Abbreviations*: No. Rec**,** number of occurrence records after spatial rarefication by a 5-km resolution**;** MTSS, the logistic threshold of maximum training sensitivity plus specificity; AUC, the area under the receiver operating characteristic curve; Bio_2, mean diurnal range in temperature; Bio_3, isothermality (daily/ annual temperature range); Bio_5, maximum temperature of warmest month; Bio_7, temperature annual range; Bio_11, mean temperature of coldest quarter; Bio_14, precipitation of driest month; Bio_15, precipitation seasonality; Bio_16, precipitation of wettest quarter; Bio_18, precipitation of warmest quarter; Bio_19, precipitation of coldest quarter.
