# Supplementary figures and images for: Ice age unfrozen: severe effect of the last interglacial, not glacial, climate change on East Asian avifauna
Source: BMC Evol Biol. 2017 Dec 6;17:244. doi: 10.1186/s12862-017-1100-2 (PMC5719578; doi:10.1186/s12862-017-1100-2)

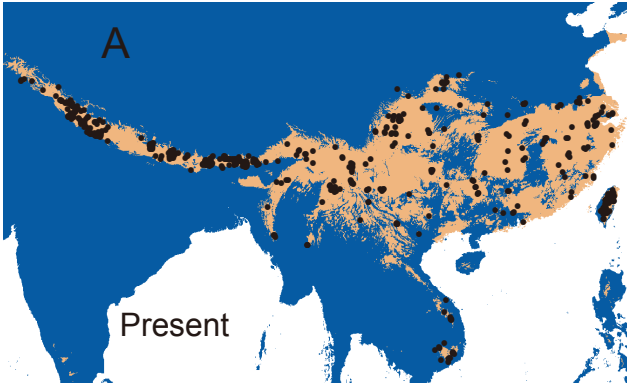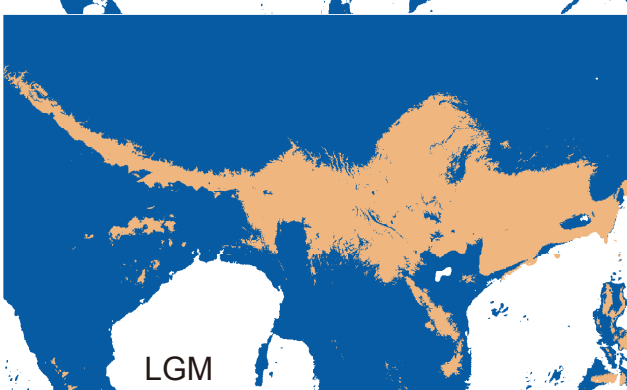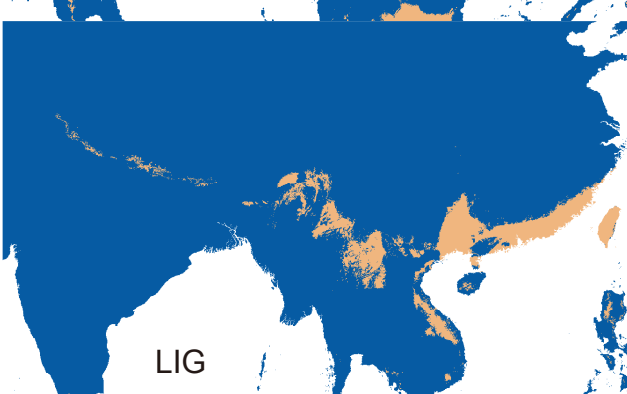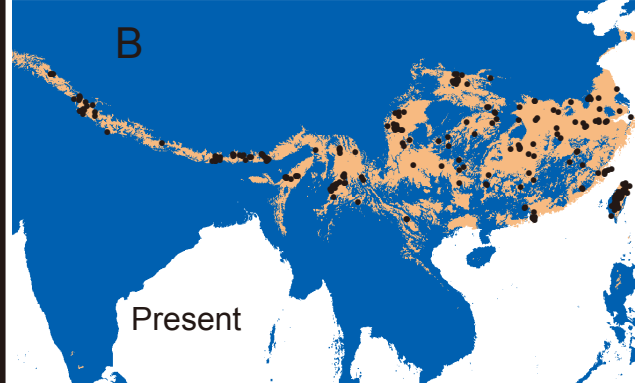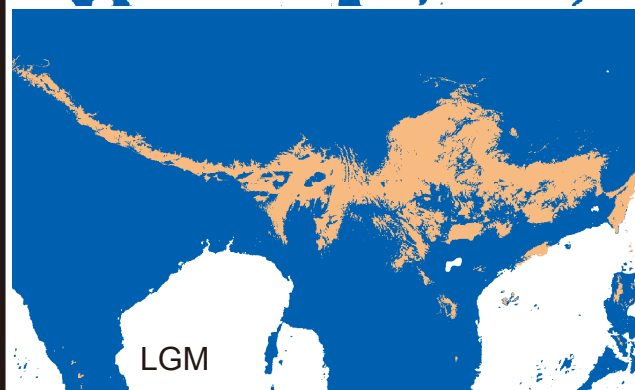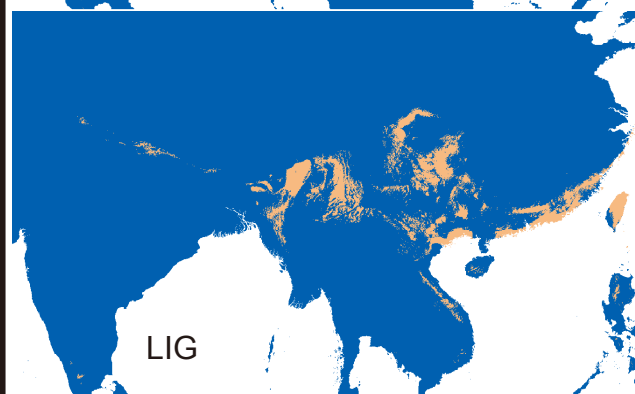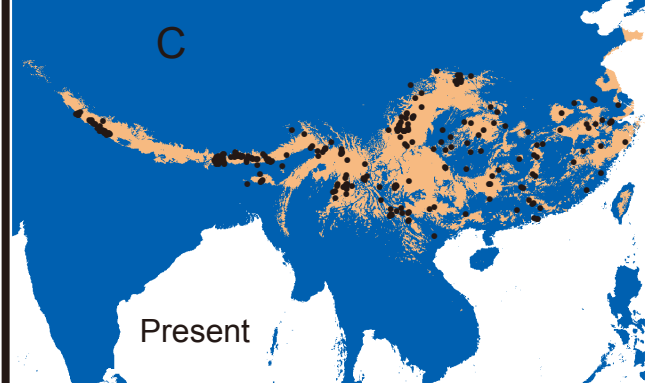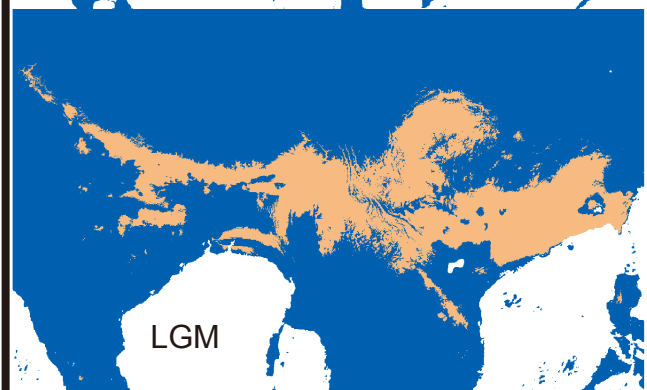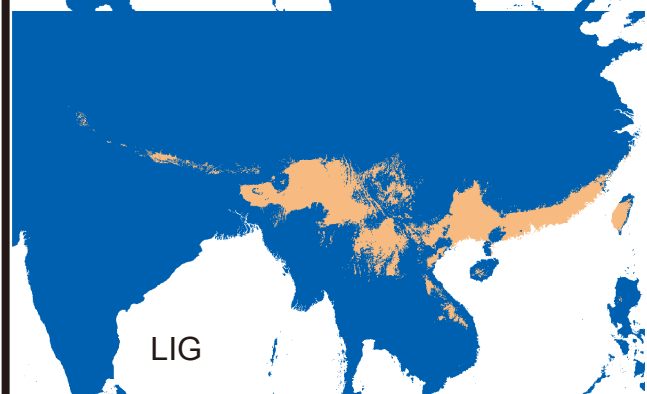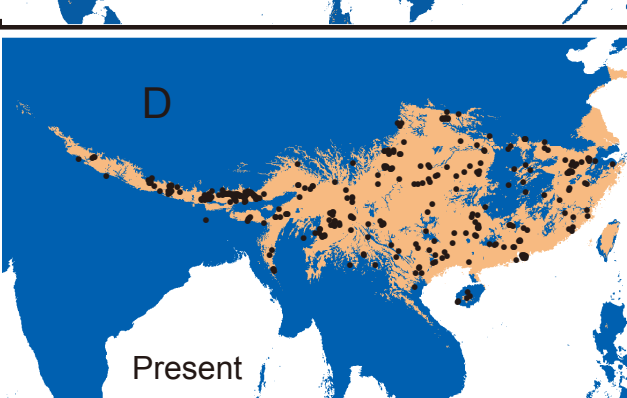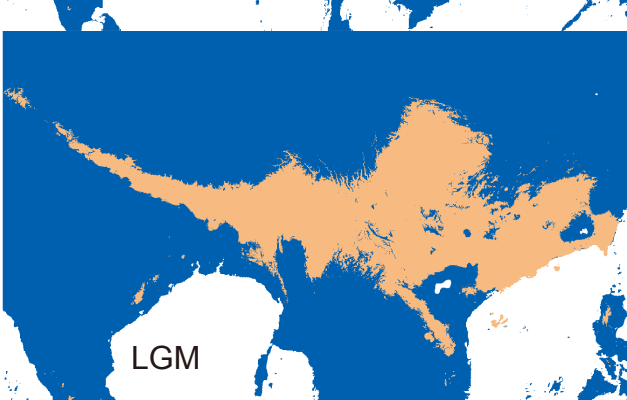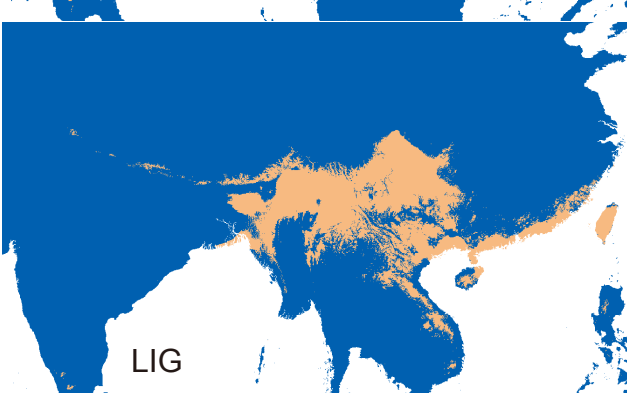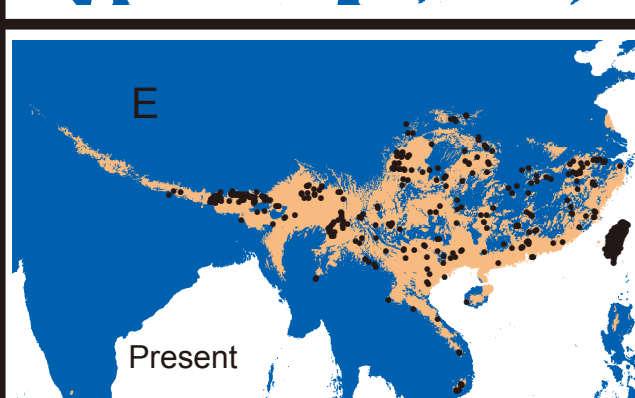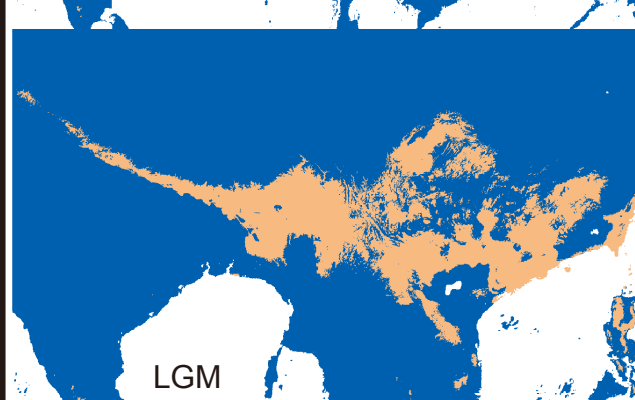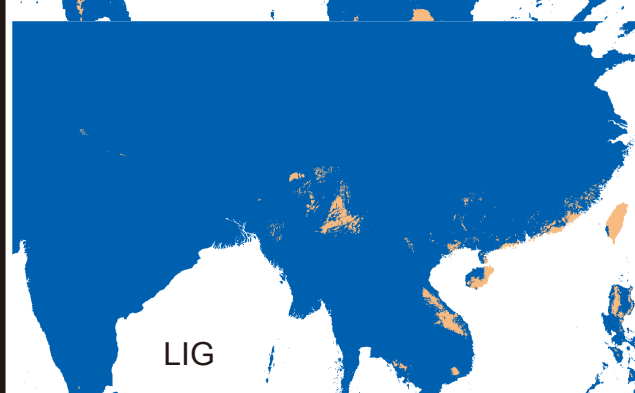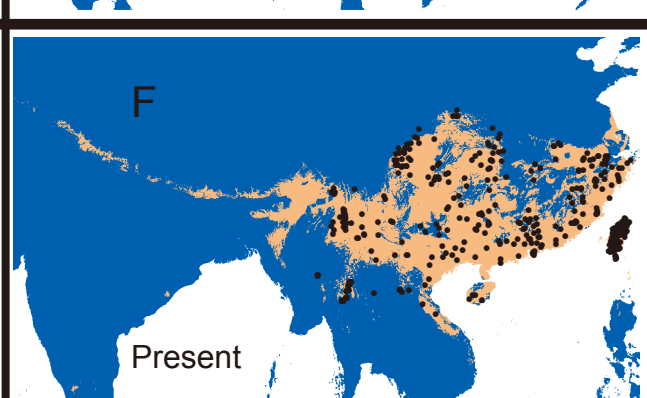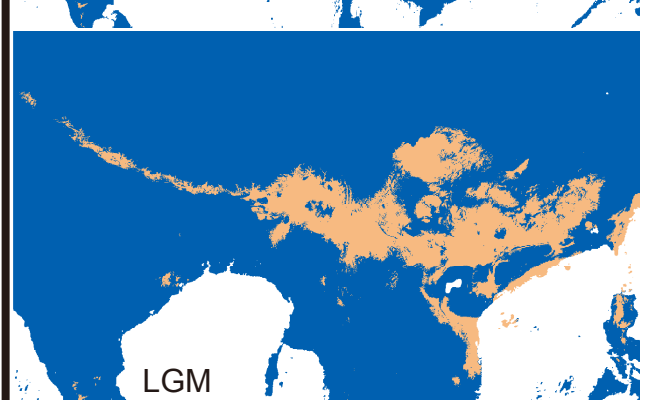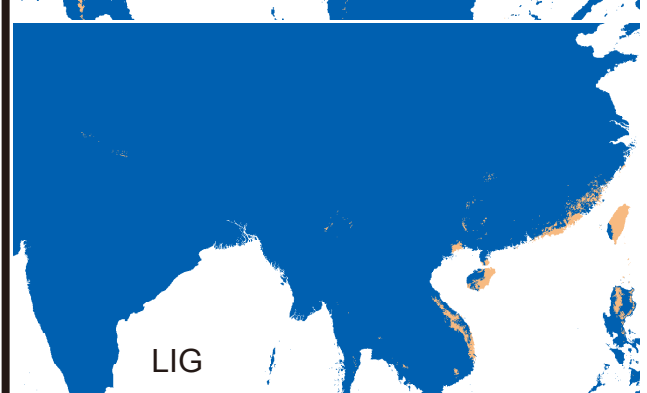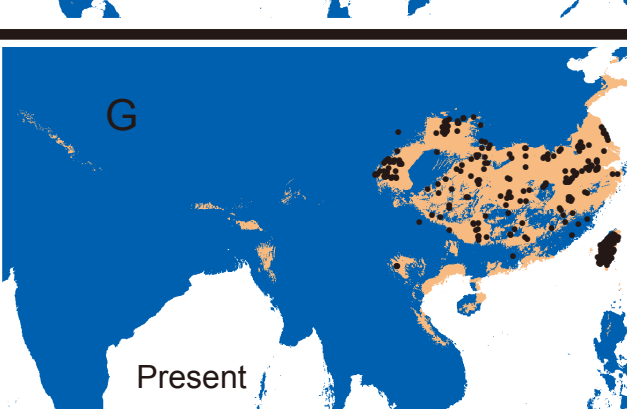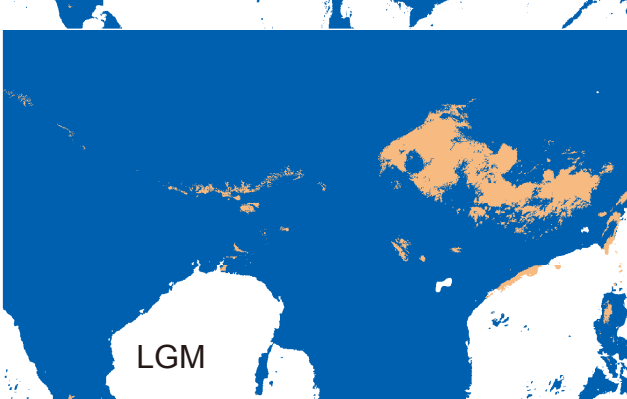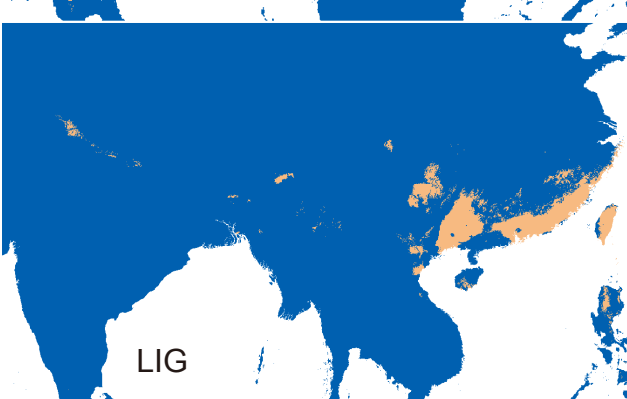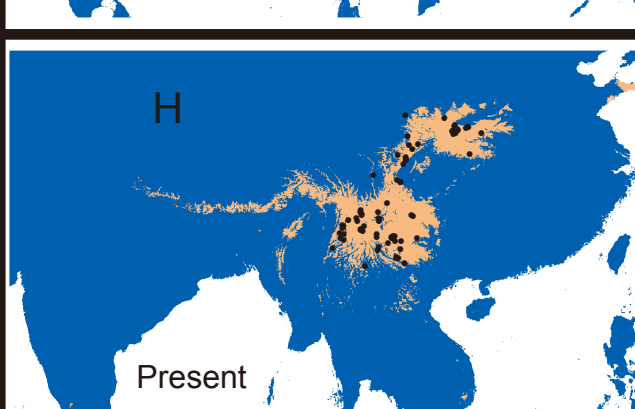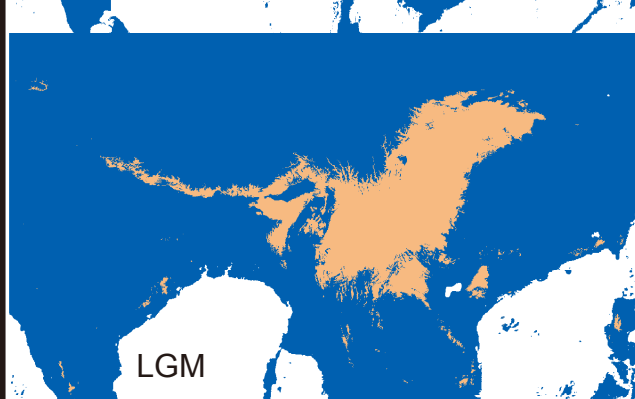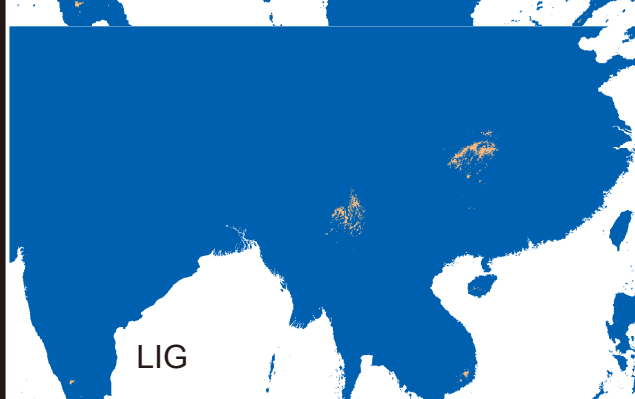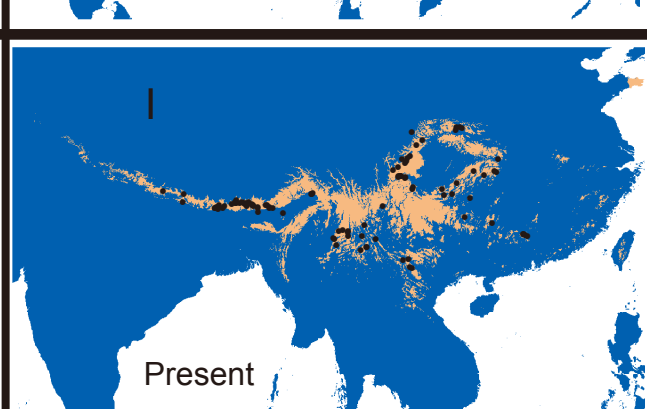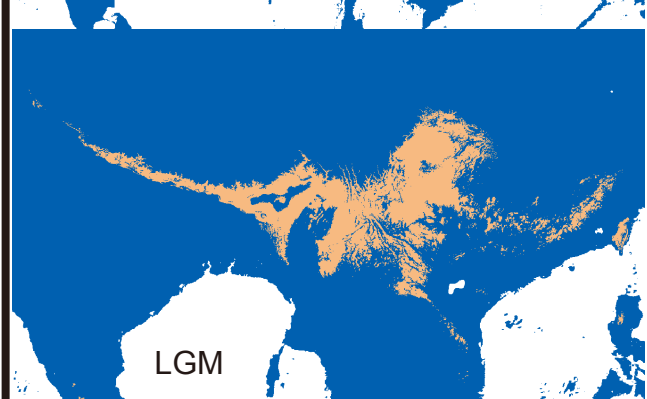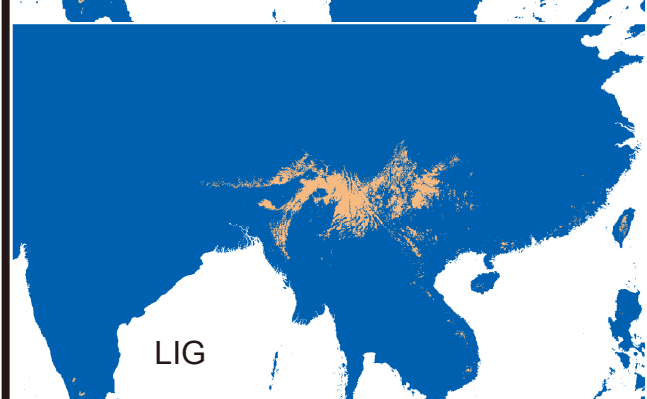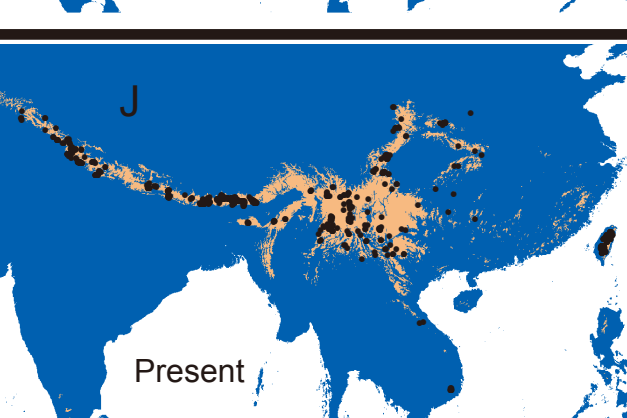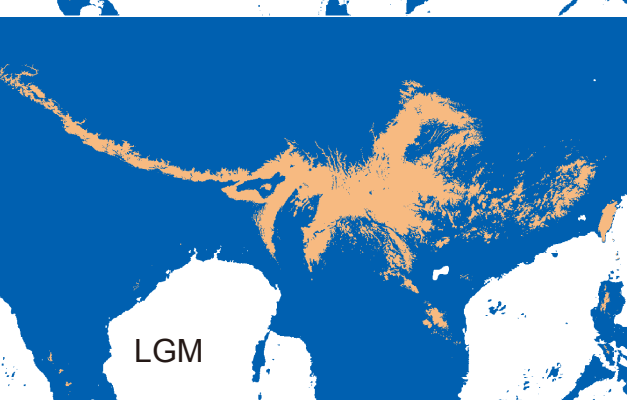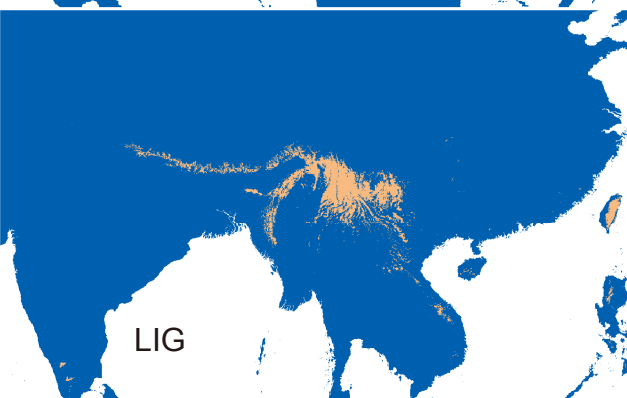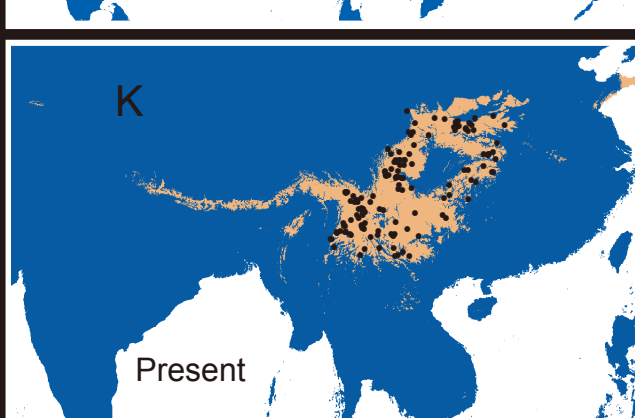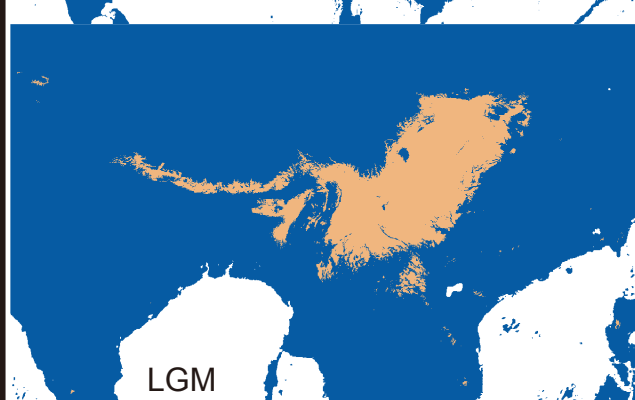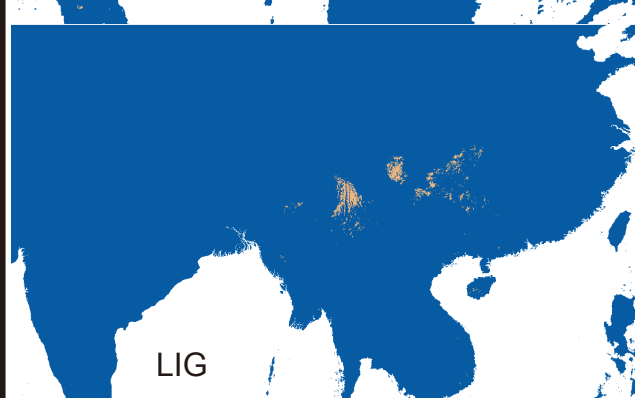

Supplement: Supplementary file 4 — Ecological niche models (ENM) and distribution dynamics. (A) Aegithalos concinnus, (B) Cettia fortipes, (C) Leiothrix lutea, (D) Pomatorhinus ruficollis, (E) Stachyridopsis ruficeps, (F) Alcippe morrisonia, (G) Spizixos semitorques, (H) Fulvetta ruficapilla, (I) Lioparus chrysotis and (J) Parus monticolus (K) Yuhina diademata. For each species, three time points were considered, i.e., the present day (Present), last glacial maximum (LGM; ~26–19 thousand years ago, Ka), and last interglacial period (LIG; ~132–112 Ka). Black dots show species occurrence records rarefied at a 5-km spatial resolution. To avoid over-prediction, the ENMs were transformed to binary (presence/ absence) distribution by a threshold of the maximum training sensitivity plus specificity. (PDF 9168 kb) [file 12862_2017_1100_MOESM4_ESM.pdf]

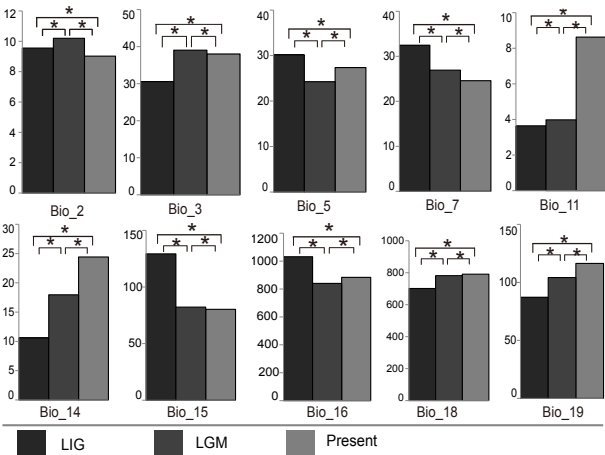

Supplement: Supplementary file 5 — Histograms indicating comparisons of ten climatic variables among LIG, LGM and Present. The means of each variable was calculated based on 1506 occurrence records after spatial filtration at 5 km of all the combination records of 11 birds. The temperature data are in centigrade (°C) and precipitation is in millimeters (mm). The asterisk (*) indicates P ≤ 0.001 calculated with a paired-samples T test. The results show larger seasonal climatic variability at the LIG than the LGM or the present, being warmer in summers (Bio_5), colder in winters (Bio_11), drier in dry seasons (Bio_14) and wetter in wet seasons (Bio_16). (PDF 407 kb) [file 12862_2017_1100_MOESM5_ESM.pdf]

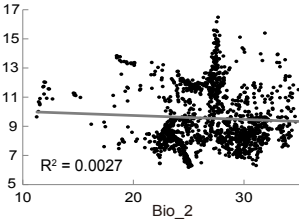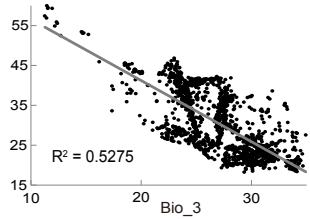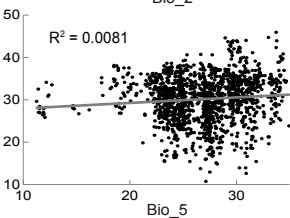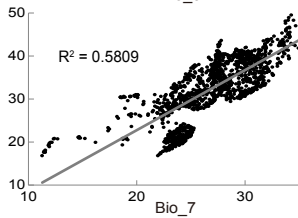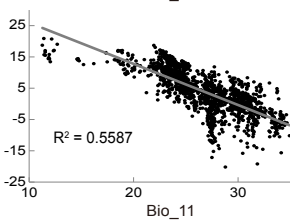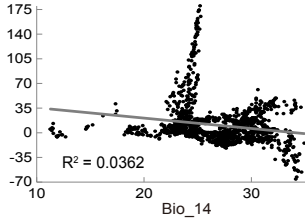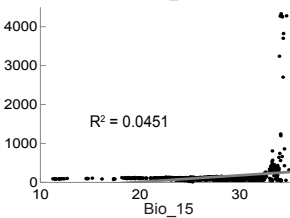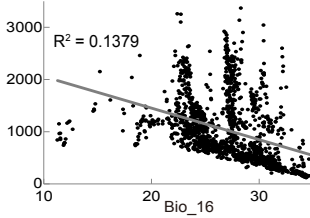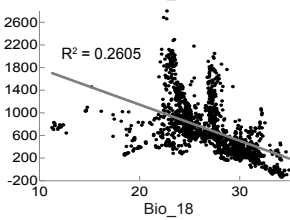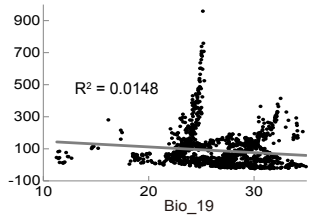

Supplement: Supplementary file 6 — Relations between climatic indices (y axis) and latitudes (x axis) at LIG. The temperature data are in centigrade, precipitation is in millimeters and the latitudes are in degrees. The results show that annual temperature variations increased while winter precipitation and temperature decreased toward the north at the LIG. (PDF 1576 kb) [file 12862_2017_1100_MOESM6_ESM.pdf]

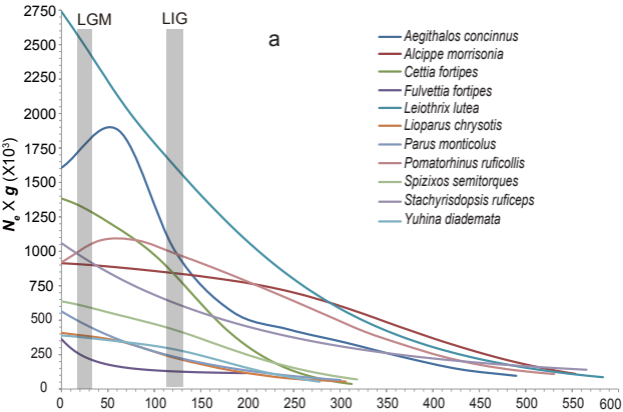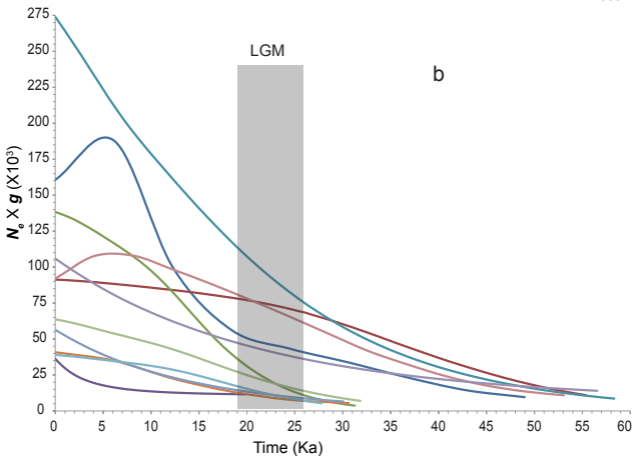

Supplement: Supplementary file 8 — Demographic reconstruction of the most favored models for each bird studied at original (a) and 10-fold (b) molecular rates. The colored plot lines represent median posterior estimates of the product of effective population size (N e) and generation time (g). Times are in thousands of years before present (Ka). Timeframes are marked by grey areas. LIG, last interglacial period, ~112–132 Ka; LGM, last glacial maximum, ~19–26 Ka. (PDF 476 kb) [file 12862_2017_1100_MOESM8_ESM.pdf]
